# High-throughput discovery of fluoroprobes that recognize amyloid fibril polymorphs

**DOI:** 10.1038/s41557-025-01889-7

**Published:** 2025-08-14

**Authors:** Emma C. Carroll, Hyunjun Yang, Wyatt C. Powell, Annemarie F. Charvat, Abby Oehler, Julia G. Jones, Kelly M. Montgomery, Anthony Yung, Zoe Millbern, Alexander I. P. Taylor, Martin Wilkinson, Neil A. Ranson, Sheena E. Radford, Nelson R. Vinueza, William F. DeGrado, Daniel A. Mordes, Carlo Condello, Jason E. Gestwicki

**Affiliations:** 1 Department of Chemistry, San José State University, San José, CA USA; 2 Institute for Neurodegenerative Diseases, University of California, San Francisco, San Francisco, CA USA; 3 Department of Pharmaceutical Chemistry, University of California San Francisco, San Francisco, CA USA; 4 Department of Biochemistry, Brandeis University, Waltham, MA USA; 5 Department of Textile Engineering, Chemistry and Science, North Carolina State University, Raleigh, NC USA; 6 Astbury Centre for Structural Molecular Biology, School of Molecular and Cellular Biology, Faculty of Biological Sciences, University of Leeds, Leeds, UK; 7 Department of Pathology, University of California San Francisco, San Francisco, CA USA; 8 Department of Neurology, University of California San Francisco, San Francisco, CA USA

**Keywords:** Screening, Sensors and probes

## Abstract

Aggregation of microtubule-associated protein tau into conformationally distinct fibrils underpins neurodegenerative tauopathies. Fluorescent probes (fluoroprobes) such as thioflavin T have been essential tools for studying tau aggregation; however, most of them do not discriminate between amyloid fibril conformations (polymorphs). This gap is due, in part, to a lack of high-throughput methods for screening large, diverse chemical collections. Here we leverage advances in protein-adaptive differential scanning fluorimetry to screen the Aurora collection of 300+ fluoroprobes against multiple synthetic fibril polymorphs, including those formed from tau, α-synuclein and islet amyloid polypeptide. This screen—coupled with excitation-multiplexed bright-emission recording (EMBER) imaging and orthogonal secondary assays—revealed pan-fibril-binding chemotypes, as well as fluoroprobes selective for fibril subsets. One fluoroprobe recognized tau pathology in ex vivo brain slices from Alzheimer’s disease and rodent models. We propose that these scaffolds represent entry points for developing fibril-selective ligands.

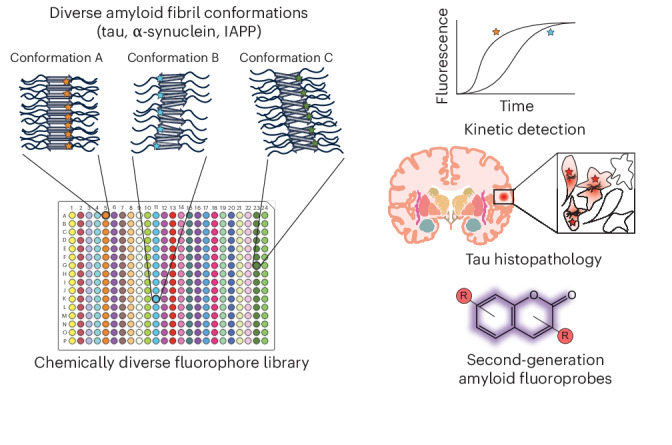

## Abstract (web summary)

Amyloid fibrils can adopt a range of distinct conformations, yet it is challenging to rapidly discriminate between these polymorphs. Now methods have been developed to screen large, diverse libraries of turn-on fluorescent dyes to rapidly identify probes that recognize fibril subsets.

## Main

Tau is an intrinsically disordered, microtubule-binding protein that assembles into β-sheet rich fibrils within the neurons of patients suffering from a family of devastating neurodegenerative diseases, known as tauopathies^1–3^. In Alzheimer’s disease—one of the most common tauopathies—these tau fibrils are characterized as either straight filaments or paired helical filaments^4^, suggesting that the same protein might be able to form fibrils with distinct molecular structures. Indeed, cryo-electron microscopy experiments have revealed that the core structure of patient-derived tau fibrils adopts different molecular conformations or folds^5^, referred to here as polymorphs. Interestingly, these fibril polymorphs seem to be disease-specific; tau fibril structures differ between some clinically distinct tauopathies but are recapitulated in patients with the same disease^6^. Together, these observations have driven interest in the development of optical reagents that can rapidly discriminate between tau fibril polymorphs.

Organic dyes have for many decades been essential tools for studying amyloid fibrils^7,8^. The power of these reagents is that their spectral properties change when they are bound to fibrils, making them relatively straightforward to use. For example, the most widely used fluorescent probe (fluoroprobe) is thioflavin T (ThT) and its fluorescence intensity is dramatically increased when bound to amyloids^7,9–12^. Likewise, fluoroprobes based on Congo Red, curcumin, polythiophenes and other scaffolds^13–18^ have proven to be convenient tools for studying how fibrils form in vitro and in cells and tissues. Fluoroprobes have also been used as competitors to identify non-fluorescent compounds by displacement^19^. Although fluoroprobes have played critical roles in studying tauopathies, they typically lack specificity for different fibril polymorphs^20,21^. Indeed, their generality is often a great strength because a single fluoroprobe such as ThT has the versatility to detect a wide range of fibrils, largely independent of sequence or substructures. Yet the field would also benefit from complementary fluoroprobes that are selective for subsets of tau polymorphs.

Most amyloid ligands have been generated by creating close structural analogues of established amyloid-binding scaffolds such as ThT or curcumin^14,22,23^. Although those efforts are often successful in producing analogues with improved properties such as brightness or permeability, they do not typically involve sampling of a wide range of chemical space. We hypothesized that more diverse starting points might be uncovered by screening larger dye collections containing a greater variety of chemical scaffolds. We saw an opportunity to address this persistent challenge in the recent development of a protein-adaptative differential scanning fluorimetry (paDSF)-based platform that leverages the Aurora collection of 300+ chemically diverse dyes^24^. To test this idea, we produced tau fibrils formed from either wild type (WT) or the P301S point mutation in *MAPT*. This mutation is linked to frontotemporal dementia; our data, and the work of others^25–28^, have shown that tau containing the P301S (or the related P301L) mutation feature distinct fibril structures. To further diversify the structure(s) of these fibrils, we also varied the polyanion used to induce in vitro tau aggregation reactions, as it has recently been shown that the identity of the polyanion inducer also contributes to fibril structure^29–31^. We then screened each of these fibril samples against the Aurora collection using fluorescence-based paDSF in 384-well plates and validated the resulting dye hits using two orthogonal secondary assays: multidimensional spectral confocal microscopy and kinetic aggregation assays.

Using this workflow we found that a subset of the hit molecules bound most of the tau polymorphs (that is, pan-fibril binders), whereas others were relatively specific to subsets of fibril conformers (that is, selective fibril binders). These molecules included compounds with coumarin and polymethine scaffolds, chemotypes that are under-represented in the field of amyloid-binding dyes^32,33^, as well as chemotypes not previously associated with amyloid recognition. To demonstrate the generality of this screening workflow, we also performed paDSF assays on fibrils composed of α-synuclein and islet amyloid polypeptide (IAPP), again revealing both pan-fibril binders and more selective tools. To show the utility of this approach in finding useful fluoroprobes, we validated one hit molecule for histological staining of tau deposits in ex vivo brain slices from a transgenic mouse model of tauopathy and post-mortem samples from patients with Alzheimer’s disease. We therefore envision that this paDSF-enabled workflow could be adapted for discovery of fluoroprobes that recognize disease-specific fibril conformers, for tau and other amyloid-prone proteins.

## Results

### Tau fibril polymorph collection for screening

**Fig. 1:**
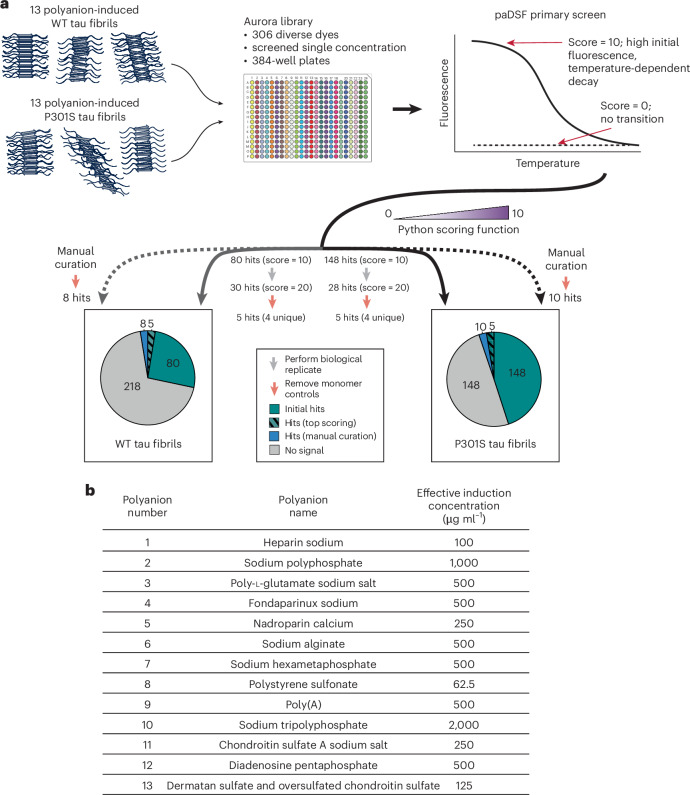
A high-throughput screening platform reveals fluoroprobes that recognize tau fibril polymorphs. **a**, Schematic of the primary screening workflow and summary of the results. Synthetic tau fibrils with diverse conformations were generated using either recombinant WT or P301S tau (0N4R splice isoform) mixed with 13 different inducers (see (**b**) and Supplementary Table 1). These 26 fibril samples were purified and incubated with the Aurora dye library in 384-well plates and then heated to generate temperature versus fluorescence plots. The resulting data were scored using a Python-based function (see Methods), with the top hits (score = 10) being dyes with high initial fluorescence, low background in the control (polyanion inducer; no tau) and a temperature-dependent decay. The highest-scoring hits across two biological replicates (cumulative score = 20) were then compared with the second control (monomeric tau alone; salmon-coloured arrows), yielding five hits that reacted with at least one of the WT or P301S fibrils (hashed green). This list was supplemented by manual curation of other top performing dyes (blue). **b**, List of the polyanions used to further diversify the fibril conformations and the concentration at which they were used.

Our goal was to establish a platform for rapid discovery of conformationally selective, fibril-binding fluoroprobes (Fig. 1a). We focused on tau as an initial model, as this protein has been shown to adopt a large number of fibril polymorphs^5^. First we needed to produce tau polymorphs at quantities sufficient for screening (that is, micrograms to milligrams). Although fibrils isolated from patients with tauopathies—such as Alzheimer’s disease and progressive supranuclear palsy—are known to have distinct polymorphs^5^, the expected sample demands led us to consider alternative sources. Synthetic fibrils made in vitro from recombinant, human tau containing the disease-associated mutation, P301S, are known to have a different structure from those made with WT tau^27^. Moreover, recombinant tau can also be coaxed into distinct polymorphs by replacing the salt^34^ or polyanion^29^ component of the buffer during the aggregation reaction. For example, polyanions such as heparin or polyphosphate are typically required for tau fibril formation in vitro and the identity of the polyanion has a substantial impact on the conformation of the resulting tau fibrils, as judged by transmission electron microscopy and limited proteolysis^29–31^. Thus, to develop a workflow for discovery of polymorph-specific fluoroprobes, we expressed and purified recombinant human tau with the 0N4R splice variant, as either WT or the P301S point mutation. These proteins were then mixed with 13 different polyanions (Fig. 1b and Supplementary Table 1) in aggregation reactions, yielding a total of 26 fibril samples. Partial proteolysis experiments confirmed that the fibrils sample a range of conformations (Supplementary Fig. 1). Although we do not expect that these synthetic fibrils will have the same structure as patient-derived samples^35^, they have the practical advantages of being scalable.

### High-throughput screen to identify tau fibril fluoroprobes

The fluorescence intensity of fluoroprobes such as ThT is often dramatically increased in the relatively hydrophobic and rigid environment of an amyloid fibril^7,9–11^. We therefore envisioned that fluorescence intensity would be a good surrogate for fibril binding in the protein-adaptive differential scanning fluorimetry (paDSF) platform. Briefly, psDSF combines a next-generation data analysis pipeline with the Aurora collection of organic dyes to probe changes in the interaction(s) between dyes and proteins during heating^36^. The Aurora library is composed of 306 chemically diverse compounds, including those typically used in laser manufacturing, the textile industry, biological imaging and other diverse applications (refer to ref. ^37^ for a full list)^36^. This collection also features compounds from the Max Weaver dye library collection (MWC), which has demonstrated high chemical diversity^38^. Speed is another key feature of the paDSF platform: a full screen on ~70 µg of purified protein in a standard qPCR instrument can be completed in ~2 h. Finally, fluorescence is monitored in six distinct channels over a continuous temperature ramp from 25 to 95 °C, which allows detection of changes in either wavelength and/or intensity. We therefore envisioned combining paDSF with the 26 purified tau fibril samples to identify Aurora dyes that recognize fibrils (see Fig. 1), with a special focus on those compounds that might discriminate between subsets of samples (for example, WT versus P301S).

**Fig. 2:**
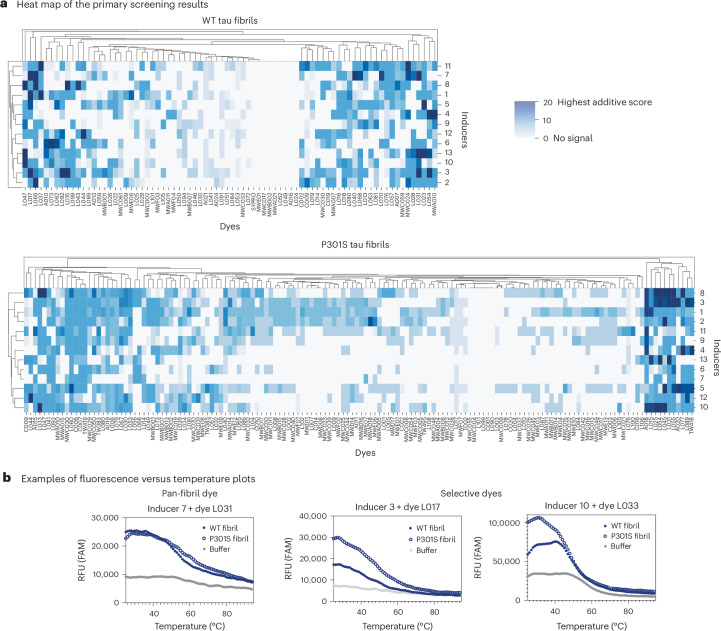
High-throughput screen results uncovers fluoroprobes that interact with either WT or P301S tau fibril polymorphs. **a**, Hierarchically clustered heat map of the additive scores for screens performed using WT (top) or P301S (bottom) tau fibrils. Only the highest scoring fluoroprobes (score = 20) were taken forward for validation. The presentation of hierarchical clustering was performed using seaborn^68^. **b**, Representative plots of relative fluorescence units (RFU, channel denoted per plot) versus temperature, highlighting the appearance of potentially pan-fibril dyes (L031) and potentially P301S-selective dyes (L017 and L033). FAM, fluorescein channel. Graphs are representative of the two biological replicates. Refer to ref. ^37^ for the full dataset and Fig. 1b and Supplementary Table 1 for a list of inducers.

In these screens (Fig. 2) we expected hits to be dyes that exhibit high initial fluorescence, indicative of binding to the fibril (see Fig. 1). We also reasoned that promising hits might display a temperature-dependent decay in fluorescence as the putative binding site(s) are impacted by heating. As controls, we counter-screened the same dyes against polyanions alone (that is, no protein) or the tau monomers alone (that is, no inducer) (Supplementary Fig. 2). Together, these screens and counter-screens produced large amounts of fluorescence versus time data, so we developed a Python-based scoring function to automate the analysis. Briefly, this scoring function assigns a score of 10 to those hits with the best signal-to-noise ratio compared with the controls (see Methods and ref. ^37^ for the Python code and raw tau screening data). We considered a score of 10 to be a rather rigorous measure of an interaction (see below).

To identify hits, we first compared the results of the fibril screens with those of the corresponding polyanion-only controls (see Fig. 1). Using this approach, the WT fibrils yielded 80 initial hits with a score of 10 (26%) across all the fibril samples, whereas screening of the P301S tau fibrils yielded 148 hits (48%). We then performed a biological replicate for each initial hit (that is, testing fibrils formed on a different day). We considered this step to be especially important because amyloid fibrillization reactions can sometimes yield variable outcomes, such as the extent of fibril formation. Indeed, the biological replicate screens turned our focus to the 30 hits (9.8%) that scored a ten in each of the two WT screens, and the 28 hits (9.1%) that scored a ten in each of the P301S fibril screens (Figs. 1 and 2a). From these replicate hits, we then removed any dyes that also interacted with the tau monomer controls, resulting in five hits (1.6%) from each of the WT and P301S screens. One of these dyes was shared between the screens, suggesting the potential for pan-fibril binding (see below). Then, to supplement this list, we manually re-analysed the primary screening results to identify dyes that scored just below the cutoff but had other favourable properties (for example, low intrinsic fluorescence, diverse chemical structures or particularly favourable initial results that did not fully replicate; see Methods). Indeed, after a parallel replication and triage process to remove monomer-active compounds, we identified a further eight validated hits (2.6%) from the WT screening and 10 (3.3%) from the P301S screening. Thus, this primary screening and replication effort yielded 27 total fluoroprobes that interacted with at least one of the tau fibrils (Fig. 1 and Supplementary Table 2); this list seemed to include both pan-fibril binding and potentially selective molecules.

A closer inspection of the raw data supported the accuracy of the computational analysis pipeline. For example, dye L077 only bound to P301S tau fibrils and not WT fibrils, especially in the presence of polyanion 4 (Fig. 2b), making it an example of a fluoroprobe with promising selectivity. By contrast, some of the fluoroprobes seemed to be more general fibril binders; for example, the dye L033 bound both WT and P301S tau fibrils that had been incubated with polyanion 10 and the dye L031 bound both WT and P301S tau fibrils incubated with polyanion 7 (Fig. 2b). This primary screen therefore revealed both putatively pan-specific fluoroprobes and ones that seemed relatively selective (that is, did not bind all of the fibril samples).

### Validation of selective fluoroprobes

**Fig. 3:**
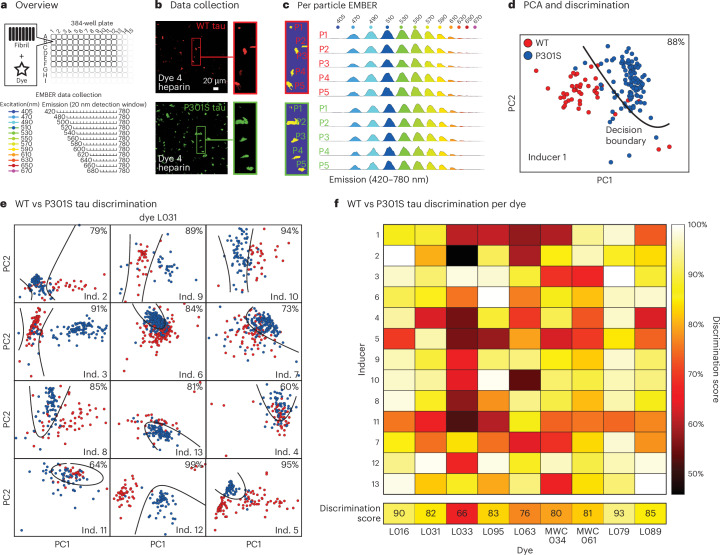
EMBER analysis suggests that fluoroprobes bind in distinct chemical environments between WT and P301S tau fibrils. **a**, Overview of the EMBER workflow. In the initial screen, the 27 hit dyes from the primary screen (see Fig. 2) were screened against twelve synthetic tau fibril samples in 384-well plates. In EMBER, fluorescence data are collected at a range of excitation and emission wavelengths to explore shifts in either the wavelength or intensity after dye binding to fibrils. **b**, Representative EMBER results, showing individual tau fibril particles composed of either WT tau (red) or P301S tau (green). Insets show the same particles at higher resolution. This example shows WT and P301S tau fibrils with inducer 1 and dye L031. **c**, For each particle, Bradley–Roth segmentation is performed across the full wavelength range to provide the EMBER plot. The particles are the same as in **b**. **d**, Individual EMBER plots are then concatenated for PCA, followed by quadratic discrimination to quantify polymorph selectivity. In this case, dye L031 was able to discriminate between WT and P301S tau fibrils (in the presence of inducer 1) with 88% accuracy. Boundaries pertaining to the fit discriminants are represented by the black line. PC1, principle component 1; PC2, principle component 2. **e**, Representative data showing that dye L031 is also able to discriminate between WT and P301S tau fibril samples created using other inducers (Ind). **f**, Discrimination heatmap for the full dataset, showing that a subset of the dyes can discriminate between tau fibril samples.

To validate the hits from the psDSF screen using an orthogonal measurement, we employed our recently developed method known as excitation-multiplexed bright-emission recording (EMBER) imaging—a multidimensional fluorescence spectral confocal microscopy technique that enables the detection of different tau fibril strains on the basis of shifts in excitation and emission spectra following dye binding^39^. First, the 27 hit fluoroprobes were tested for direct binding to a subset of fibrils via fluorescence confocal microscopy. In these pilot experiments we used only a subset of synthetic fibrils (WT and P301S tau, plus six different inducers; see Methods) because the subset was predicted by the primary screen to include polymorphs that would putatively cover the entire dye set. From these microscopy experiments, 10 out of the 27 dyes (37%) were found to bind at least one of the fibrils (Fig. 3a). Next, we subjected these ten dyes to the more complete EMBER imaging pipeline. Briefly, the ten fluoroprobes were mixed with each of the 26 tau fibril samples and imaged with confocal microscopy (Fig. 3a). For each dye–fibril pair, we collected fluorescence information for tau fibril particles (Fig. 3b), using a white light laser to collect 128 spectral profiles across the visible spectrum (Fig. 3c). We then segmented the tau fibril particles from the confocal images and concatenated the excitation and emission profiles in an array for principal component analysis (PCA). Using this approach, each marker on the resulting PCA plot represents a single segmented particle in a confocal image (Fig. 3d). Next we investigated whether any of the hit dyes could discriminate between WT and P301S tau fibrils by examining the separation of the corresponding particles in the PCA plot. To quantify this cluster separation between WT and P301S tau in the presence of each inducer, we employed quadratic discriminant analysis on each PCA plot to calculate a discrimination score. Satisfyingly, this process identified fluoroprobes with the ability to discriminate between the polymorphs. For example, comparing WT versus P301S tau fibril with inducer 12, we found that dye L031 yielded a clear decision boundary from the quadratic discriminant analysis (Fig. 3e). We averaged the discrimination scores comparing WT and P301S tau fibrils across the 13 inducers to provide an overall discrimination score for each fluoroprobe. The heatmap (Fig. 3f) shows that the discrimination scores ranged from 93% to 66%, with L079 and L016 having the highest overall values. Based on these data, we speculate that WT and P301S tau fibrils remain partially distinct, even if the identity of the polyanion is changed, but this hypothesis requires additional validation. Refer to ref. ^37^ for the complete EMBER datasets. Together, these studies show that EMBER is a valuable secondary assay, which we used to confirm fluoroprobes that discriminate between WT and P301S tau fibril polymorphs.

### Applying screening to other fibril-forming proteins

Based on the success of the paDSF screening platform at identifying tau fibril-binding fluoroprobes, we next sought to test whether this method might be more generally applicable to fibrils formed from other proteins. Towards this goal, we produced and purified WT α-synuclein and IAPP. Both of these proteins readily form fibrils in vitro and their conformations have been explored^40,41^. We also purified the S20G mutant of IAPP because it has been shown to produce distinct polymorphs from the WT^41,42^. Based on the results of the tau screens, we reasoned that screening fibrils formed from these proteins might also yield both shared and unique hits. After preparing these three fibril samples (see Methods), both the IAPP samples were screened and analysed using the same computational pipeline as used for tau fibrils, whereas α-synuclein was screened with an abbreviated procedure in which fluoroprobe hits were scored manually (see Methods). Refer to ref. ^37^ for the raw screening data from the synuclein and IAPP assays.

**Fig. 4:**
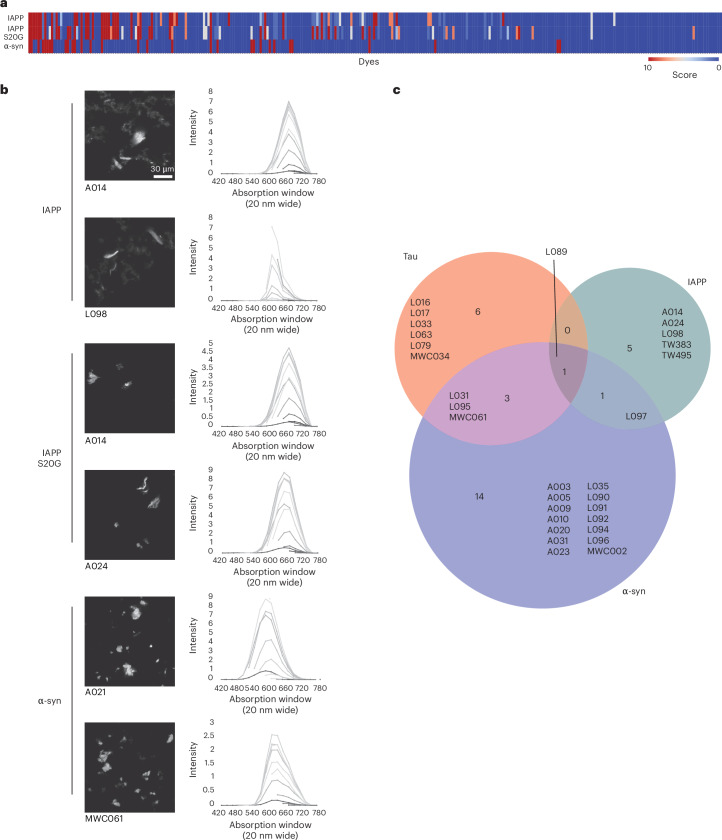
paDSF fluoroprobes display selective recognition for diverse amyloid-forming proteins. **a**, Heat map showing pDSF screening results for WT and S20G IAPP fibrils and WT α-synuclein fibrils. IAPP paDSF screens were performed and results were scored as described for tau fibrils, whereas α-synuclein fibrils were screened with an abbreviated procedure using manual scoring. **b**, Selected confocal micrographs (left) and EMBER profiles (right) validating fluoroprobe binding to WT and S20G IAPP fibrils and α-synuclein. **c**, Venn diagram summary of paDSF screening data for tau (all EMBER-validated inducer fibril types combined), IAPP (WT and S20G combined), and α-synuclein fibrils. Each amyloid-forming protein exhibits both unique and shared hits, with only one fluoroprobe hit shared between all three proteins.

The α-synuclein paDSF screen yielded 33 preliminary hits, whereas the IAPP screens yielded ten preliminary hits (Fig. 4a). Each of these molecules were then tested for direct binding by confocal microscopy. This triage process revealed that 19 of the 33 α-synuclein fluoroprobe hits and seven of the ten IAPP fluoroprobe hits were positively identified as binding to fibrils (Fig. 4b and Supplementary Figs. 3 and 4). Satisfyingly, this confirmation rate approximated the value observed in the tau screen. Interestingly, only one hit, L089, was shared between all three amyloid-forming proteins (that is, tau, α-synuclein and IAPP). Moreover, α-synuclein and IAPP fibrils shared only one validated hit, whereas tau and α-synuclein shared three hits. No hits were shared between tau and IAPP (Fig. 4c). These results suggest that the paDSF screening platform is generalizable to diverse amyloid-forming proteins, with the capacity to rapidly generate fibril-specific hits that may recognize unique sites.

### Screening reveals underexplored chemotypes

**Fig. 5:**
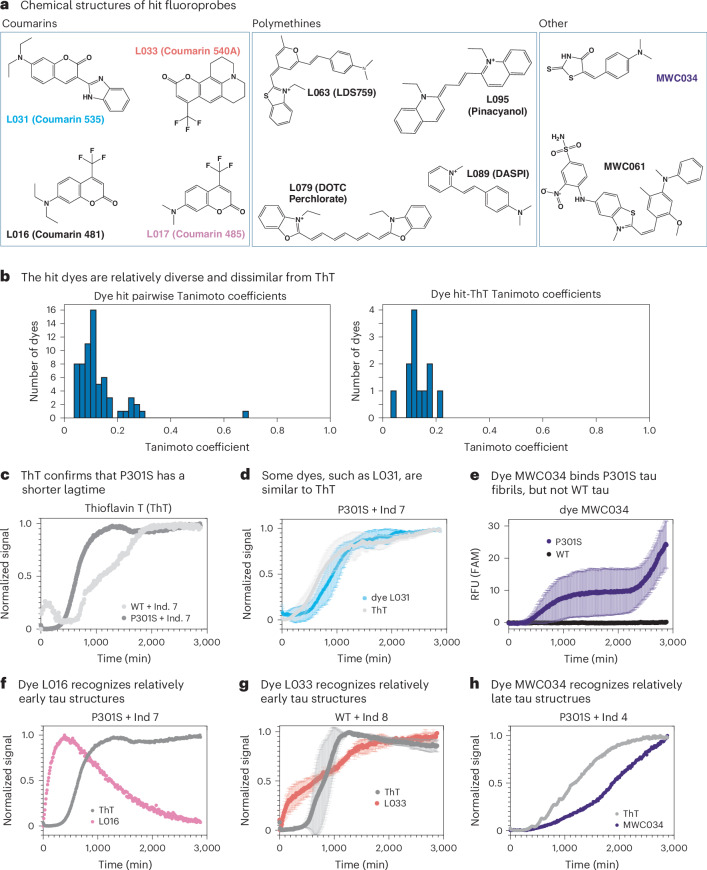
Validated fluoroprobe hits are chemically diverse and can detect tau fibril formation in real-time. **a**, Chemical structures of the validated fluoroprobe hits, showing the two clusters (coumarins and polymethines). **b**, Histogram of pairwise Tanimoto similarities for all hits compared with one another (left), and with ThT (right). Tanimoto coefficients were calculated using a script created with the RDKit Python package (see ref. ^37^). **c**–**h**, Kinetic aggregation assays. Either ThT (10 µM) or the hit dyes (50 µM, except for L031, which was 0.5 µM) were mixed with WT or P301S tau (10 µM), and aggregation was initiated with a polyanion inducer. Raw signal was normalized as a fraction of total signal to fall between 0 and 1 to facilitate comparisons. Data points are from single, representative experimental curves. **c**, Confirmation that P301S aggregates faster than WT, as shown using ThT and inducer 7. **d**, Example of a dye, L031, that has a similar profile to ThT. P301S tau with inducer 7 is shown. **e**, Example of a dye, MWC034, that only recognizes P301S tau, and not WT. Reactions contained inducer 7. **f**, Example of a dye, L016, that recognizes structures early in the process than ThT. Results from P301S and inducer 7. **g**, L033 recognizes relatively early structures, using WT tau and inducer 8. **h**, Dye MWC034 recognizes relatively late structures, using P301S tau and inducer 4.

Together, the paDSF screen and EMBER confirmation assay yielded ten validated hits that bound to the tau fibrils. This list was enriched for two major chemotypes: coumarins and polymethines (Fig. 5a), but it was also relatively chemically diverse as evidenced by low Tanimoto pairwise similarity coefficients when compared with each other (Fig. 5b, left). These molecules also had relatively poor Tanimoto pairwise similarity coefficients when compared with ThT (Fig. 5b; right) or nine other known tau-binding probes (Supplementary Fig. 5), suggesting that they diverge from the standard fluoroprobes. Indeed, both coumarins and polymethines are relatively underexplored as amyloid ligands dyes^32,33^ and the other compounds, MWC034 and MWC061, have never (to our knowledge) been reported to have amyloid-binding properties. This is an important finding because we intended for the paDSF platform to identify understudied chemotypes.

### Fluoroprobe activity in kinetic aggregation assays

As a next step in validating these probes, we used them in ThT-like kinetic aggregation assays to monitor fibril formation. One goal of these experiments was to test whether any of the dyes could also be used to monitor fibril formation over time. This was important because both the psDSF and EMBER platforms focus on end-stage fibrils, and we hypothesized that the fluoroprobes may detect structures that appear relatively early or late in the tau aggregation reactions in vitro. In these experiments, WT or P301S tau protein was treated with eight different polyanions and the reactions shaken in an incubator in the presence of either ThT or one of the ten confirmed fluoroprobes. As expected^43^, the positive control, ThT, produced curves with a characteristic lag time, followed by maturation of the signal until a plateau is reached (refer to ref. ^37^ for the full dataset). For example, in the presence of inducer 7, both WT and P301S tau formed ThT-active fibrils (Fig. 5c). These controls also confirmed that P301S tau is more aggregation-prone than WT; the lag time was shorter for P301S tau compared with WT tau. When we replaced ThT with the hit fluoroprobes, nearly all of them (7/10, 70%) were active in this format, supporting the idea that the kinetic aggregation assay is another useful secondary screening platform. Moreover, we observed a range of interesting behaviours of the identified dyes, allowing us to learn more about their properties. For example, the fluorescence of some compounds, such as L031, largely tracked with ThT over time (Fig. 5d). This type of result suggests that L031, like ThT, might bind a wide range of polymorphs that appear during the aggregation process (for example, oligomers, fibrils), at least under these conditions. Perhaps this result is not surprising because L031, like ThT, has a putative molecular rotor pharmacophore and a shared tertiary amine (Supplementary Fig. 6). Satisfyingly, other dyes only produced a signal in the presence of P301S tau and not WT tau. For example, in the presence of inducer 7, MWC034 produced a signal for P301S tau and not WT tau (Fig. 5e). Finally, an inspection of other kinetic traces suggested that some dyes, such as L016 and L033, might recognize structures that appear relatively early in the aggregation reaction (Fig. 5f and Fig. 5g), whereas MWC034 may recognize later structures (Fig. 5h); however, this mechanistic speculation requires further study. Moreover, we note that this assay has the complexity that some fluoroprobes might inhibit aggregation or bind to tau monomer (see below). Taken together, this high-throughput workflow, coupled with a computational analysis pipeline and two distinct secondary assays, seemed to yield multiple, promising fluoroprobe hits, including chemotypes that were either underexplored or distinct from those in the amyloid-binding literature.

### Tau pathology detected in ex vivo brain sections

**Fig. 6:**
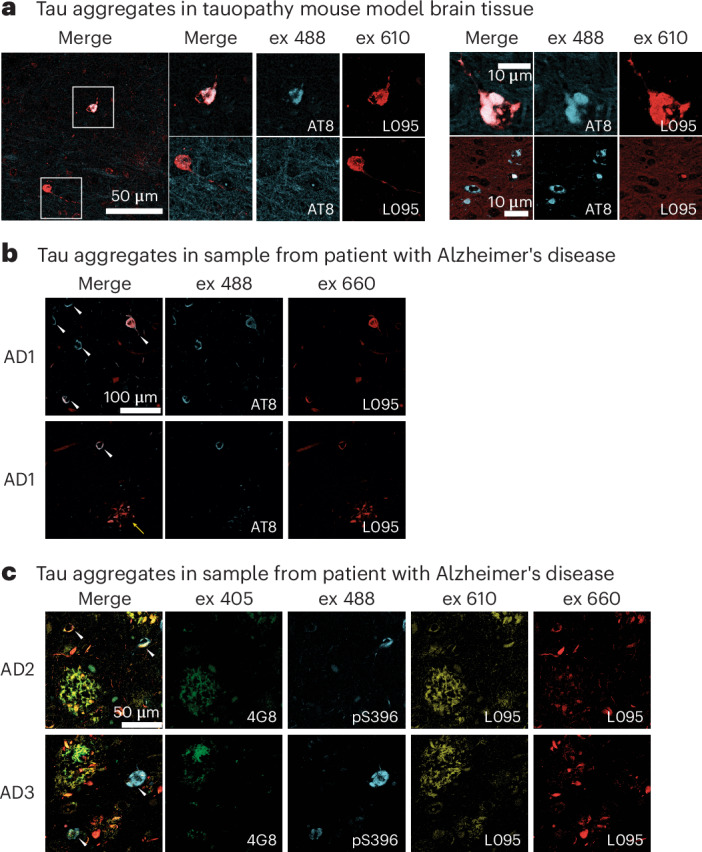
Fluoroprobe L095 recognizes tau pathology in brain tissue from mouse models and human patients with Alzheimer’s disease. **a**, Representative micrographs, at two magnifications, from hippocampal sections of Tg4510 mice expressing human MAPT (tau) containing the P301L mutation under the control of the forebrain-specific Ca^2+^/calmodulin kinase II promoter. Samples are stained with an AT8 antibody for pathological tau (blue) and L095 (red), and are also shown merged. **b**, Two samples from a patient with Alzheimer’s disease, labelled with the AT8 antibody and L095. Note that L095 recognizes tangles (white arrows), but also labels a tau pathology that is consistent with neuropil threads (yellow arrow). **c**, Samples from two patients with Alzheimer’s disease, stained with antibodies for either αβ (4G8 antibody) or tau pathology (pS396 antibody). Note that L095 co-localizes with G48 at 610 nm, but with AT8 at 660 nm, allowing spectral discrimination between the two pathologies.

One potential use of these fluoroprobes is in the detection of tau polymorphs in brain tissue, as commonly performed with Thioflavin S^44^. We therefore next tested whether any of our validated fluoroprobes might be useful in histopathological studies. As we expected that some dyes may exhibit undesirable, non-specific tissue binding properties, we pre-screened the ten hit dyes in normal mouse brain slices (lacking amyloid deposits) to remove compounds with high background fluorescence (Supplementary Fig. 7). From this pre-screen, we selected L095 as a potentially promising candidate with low background. L095 is also known as pinacyanol and it is commonly used as a laser tuning dye and was recently evaluated as a potential amyloid ligand^45^. As a proof-of-concept, we collected fixed brain slices from the Tg4510 mouse model of tauopathy^46^, which accumulates substantial cortical and hippocampal tau pathology with aging. We co-stained brain sections with L095 dye and the AT8 antibody which recognizes a hyperphosphorylated epitope present in tau deposits^29^. We observed strong L095 labelling of a subset of tau deposits with excellent contrast at 610 nm excitation and a good overlap with AT8 staining (Fig. 6a).

Following the promising results with tau deposits in the Tg4510 mouse model, we investigated whether L095 could label tau deposits in post-mortem brain samples from patients with advanced Alzheimer’s disease neuropathology. In Alzheimer’s disease samples, L095 successfully labelled tau neurofibrillary tangles with good overlap with AT8 antibody staining (Fig. 6b). Compared with the Tg4510 tau tangle staining, the optimal excitation wavelength red-shifted from 610 nm to 660 nm, suggesting the dye L095 can in situ differentiate distinct tau conformers between Alzheimer’s disease and Tg4510 samples as we have shown for other dyes^39^. This observation is in general agreement with cryo-EM studies showing distinct structures of tau filaments found in brain extracts from Alzheimer’s disease^6^ versus the Tg4510 mice^47^. Furthermore, L095 labelled tau neuropil threads, which were not robustly labelled by the AT8 antibody. Neuropil threads are morphological distinct lesions of aggregated tau found within the dendritic and axonal compartments, and can also be observed within dystrophic neurites, which are swollen, abnormal neuronal processes intermingled with senile plaque Alzheimer’s disease pathology^48^. Thus, this finding demonstrates the potential utility of L095 in staining a relatively understudied tau deposit.

As brain samples from patients with Alzheimer’s disease contain other amyloid-containing deposits such as Aβ (amyloid-β), we performed a multiplex staining experiment using the anti-Aβ antibody 4G8, the anti-tau pS396 antibody and fluoroprobe L095. First, to be certain that L095 binds to Aβ, we confirmed its interactions in brain slices from transgenic Alzheimer’s disease mice models (APP23 and 5×FAD), as well as with in-vitro-prepared Aβ40 and Aβ42 fibrils (Supplementary Fig. 8a,b). In the human Alzheimer’s disease sample, we found that L095 binds to both Aβ plaques and tau tangles, but that the fluorescence signal requires a distinct optimal excitation of 610 nm or 660 nm, respectively (Fig. 6c). This shift in excitation could be attributed to exciton coupling^49^ created by the intermolecular stacking of individual dye molecules along the fibril axis that has been observed in ligand-complexed cryo-EM structures of the amyloids, and in multispectral confocal microscopy^39,50^. Thus, L095 seems to exhibit some differential sensitivity for Aβ plaque and tau tangles in situ. As a second, independent test of the utility of the fluoroprobes from this screen, we tested a different hit, A003, in brain tissue from a patient with dementia with Lewy bodies, and confirmed that it stained α-synuclein pathology (Supplementary Fig. 9), consistent with the psDSF screening result. Refer to ref. ^37^ for the complete histopathology dataset. Although more work is needed to understand the molecular features that distinguish the L095-bound and A003-bound fibril structures, the results suggest that this in vitro to ex vivo workflow can produce fluoroprobes that stain interesting features of pathology in human brain samples.

### Possible optimization of a coumarin chemotype

**Extended Data Fig. 1:**
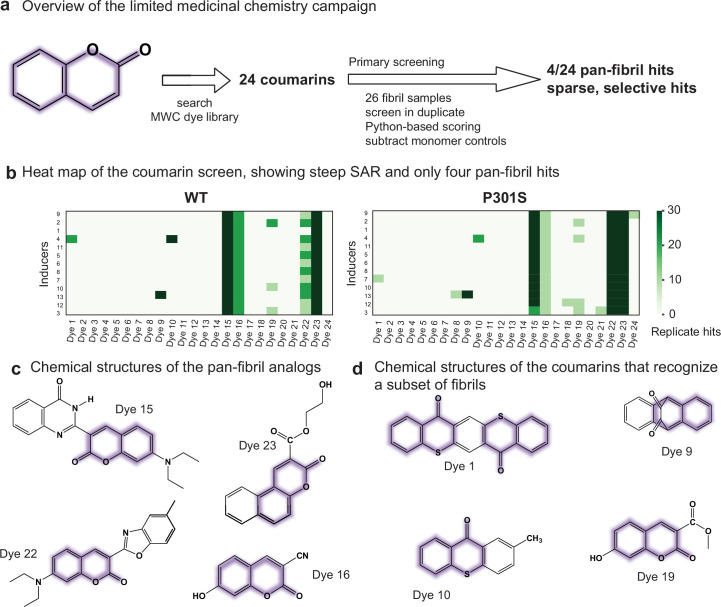
Analogues of the coumarin scaffold include both pan-fibril binding and potentially selective fluoroprobes. (**a**) Overview of the limited medicinal chemistry campaign. Twenty four coumarins from the Max A. Weaver Collection (MWC) were screened against 26 fibril samples by paDSF using a pipeline that parallels Fig. 1, except that three biological replicates were used. After triage and removal of dyes that bound tau monomer, only 4 coumarins were identified that bind both WT and P301S fibrils. (**b**) Heat maps of the screening results, showing that most of the analogues failed to recognize either the WT or P301S tau fibril samples (white); whereas a subset produced reproducible signal (green). Here, the top Python score was 30 (10 for each replicate). (**c**) The chemical structures of the coumarins that bind to both WT and P301S fibrils, suggesting that they are pan-binders. (**d**) Chemical structures of coumarins with activity against at least one WT or P301S tau fibril conformer. The coumarin scaffold is highlighted in purple.

Finally, if the fluoroprobes were binding to discrete site(s) on amyloid fibrils, we expected that a medicinal chemistry campaign might reveal structure–activity relationships (Extended Data Fig. 1a). The alternative hypothesis is that these dyes are simply sticky, such that close analogues might have similar or even identical properties. We focused on the coumarins to test these ideas. Our screening results had shown that these dyes are in the category of pan-fibril binders, meaning that they interact with both WT and P301S fibrils (see above). We therefore considered that the coumarins might potentially be sticky. To assemble an analogue set, we searched the MWC Library^21^ to select 24 additional coumarins and coumarin-related scaffolds, such as thiochromones (Supplementary Fig. 10). These compounds were then tested for interactions with the 26 WT and P301S tau fibrils in the paDSF-based primary screening platform (Extended Data Fig. 1b). Using three replicates, we found that only four dyes (15, 16, 22 and 23)—like the parent compounds—bound to both WT and P301S fibrils (Extended Data Fig. 1c). Refer to ref. ^37^ for the raw data from the coumarin analogue screen. These findings provide preliminary support to the idea that the coumarin-binding site(s) might have discrete features, rather than being non-specific or sticky. Interestingly, a subset of the analogues had more restrictive activity, only labelling either WT or P301S tau fibrils in the presence of specific inducers (Extended Data Fig. 1d), suggesting that selectively might be obtained through future efforts. Although preliminary, these findings suggest that medicinal chemistry efforts might be used to further optimize validated chemical series from the screening workflow. Further studies and analogues are needed to fully describe the structure–activity relationships or determine the properties of the binding sites on the tau fibrils.

## Discussion

Fluorescent reporters of tau fibril conformation could be used to dissect the molecular underpinnings of tauopathy and create diagnostics, such as starting points for positron emission tomography (PET) imaging probes^51,52^; however, most current fluoroprobes such as ThT bind many amyloids and do not discriminate between polymorphs. Although other groups are exploring ways to improve selectivity, such as fluoroprobes that discriminate between oligomers and fibrils^45,53–56^, we sought to supplement those efforts with a large-scale, unbiased screen of diverse organic dyes. Accordingly, we established a paDSF-based screening platform, coupled with the Aurora collection of dyes, to uncover tau fibril-binding fluoroprobes (see Figs. 1 and 2). Importantly, we expect that this platform can be leveraged to study other fibril samples of interest because over half of the compounds in the Aurora library are commercially available and screens can be performed on any standard qPCR instrument or fluorescent plate reader (see Methods).

This screen had a surprisingly low hit rate (10/306 total; ~3%), suggesting that dye binding involves discrete binding sites instead of non-specific contacts. Indeed, a limited medicinal chemistry campaign, using 24 analogues of the coumarin chemotype, generally supported this idea (see Extended Data Fig. 1). This model is also supported by classic work to define the binding sites of ThT and Congo Red^11,13^, and recent cryo-EM studies, which have shown that the PET probes, flortaucipir, APN-1607 and GTP-1, and the amyloid ligand, epigallocatechin gallate, bind to discrete sites on tau fibrils^50,57–59^. Importantly, fibril polymorphs differ dramatically in their folds and availability of pockets^60^, supporting the idea that organic dyes seem well suited to exploit the structural differences between tau fibril cores. We envision that fluoroprobes might discriminate between fibril polymorphs by two primarily mechanisms: a dye might bind to only one of the polymorphs because the binding site is only present in that target. Alternatively, the binding site might be present in both polymorphs, but the chemical environment only supports strong fluorescence increases in one of the fibril-bound forms.

Our hope is that this workflow could inspire further miniaturization for use in screening patient-derived fibrils. Alternatively, many groups are pursuing ways of generating disease-relevant synthetic fibrils in vitro^34,61,62^ that recapitulate disease polymorphs; application of our methods workflow would greatly expedite the validation and prioritization of artificial fibril-forming conditions leading to bona fide human brain-derived fibril polymorphs. Another tentative goal of this work was to show whether high-throughput approaches might be useful in revealing distinct or underexplored chemotypes in diverse libraries. Indeed, the validated hits included coumarin and polymethine scaffolds. Coumarin-containing probes are documented to have amyloid-binding properties^63–65^, but, to the best of our knowledge, none have been shown to bind tau fibrils. Likewise, polymethine and cyanine dyes, particularly those with relatively short polymethine chains^66^, have only been sporadically identified as having amyloid-binding properties^67^, but remain underexplored. Perhaps most importantly, two dyes from the Max Weaver library were identified that have not yet been reported as binding to amyloids. We therefore conclude that high-throughput approaches might be a good complement to the field’s ongoing efforts to develop polymorph-selective fluoroprobes. We hope that this technology will enable other researchers to discover fluoroprobes that bind to targets of interest, ultimately improving our molecular understanding and advancing diagnostics and therapeutics for neurodegeneration.

## Methods

### Purification of tau proteins

*Escherichia coli* BL21 Rosetta 2 (DE3) cells were transformed with pEC135 (WT 0N4R tau) or pEC146 (Tau P301S 0N4R). Cells were grown to between 0.4 and 0.8 optical density (OD_600_) in Terrific broth and then induced with 1 mM isopropyl β-d-1-thiogalactopyranoside (IPTG) for 3 h at 37 °C. Bacteria were pelleted by centrifugation (4,000 × *g*) and then resuspended in 1× distilled phosphate-buffered saline (DPBS) buffer (Corning, pH 7.4) with 10 mM EDTA, 2 mM MgCl_2_, 1 mM dithiothreitol (DTT) and 1× protease inhibitor tablets (Pierce). Resuspended cells were lysed by sonication and the lysate was clarified by centrifugation at 25,000 × g at 4 °C for 30 min. Tau variants were first purified via their *N*-terminal 6×-His tags by incubating the clarified lysate with complete His-tag purification resin (Roche, EDTA- and DTT-compatible) for 1 h at 4 °C. Bound resin was washed with 1× DPBS buffer (Corning, pH 7.4) with 10 mM EDTA, 2 mM MgCl_2_,1 mM DTT and 1× protease inhibitor tablets (Pierce) and eluted with 1× DPBS buffer (Corning, pH 7.4) with 300 mM imidazole (pH 7.4), 10 mM EDTA, 2 mM MgCl_2_,1 mM DTT and 1× protease inhibitor tablets (Pierce). Eluate was dialysed overnight at 4 °C using tau buffer: 1× DPBS buffer (Corning, pH 7.4) with 2 mM MgCl_2_,1 mM DTT. Concentration of purified protein was measured using the BCA assay (Pierce). A reverse-phase chromatography step was then performed using a Kromasil semipreparative column (250 mm length, 10 mm inner diameter, C4, 5 µm particle size, 300 Å pore size) equilibrated with 5% acetonitrile. Tau protein was eluted from the column using a 5–50% acetonitrile gradient over the course of 45 min. Fractions containing pure, full-length tau were then lyophilized to remove solvent and resuspended in tau buffer and stored at –80 °C.

### Creation and purification of tau fibrils

Aggregation reactions were performed in 1.5 ml Eppendorf tubes for 48 h at 37 °C with constant agitation at 1,200 r.p.m., which was shown to be sufficient to complete fibril formation^29^. All reactions contained 10 µM tau, plus a polyanion inducer (see below) in tau buffer (1 × DPBS buffer (Corning, pH 7.4) with 2 mM MgCl_2_,1 mM DTT) at a final volume of 300 µl. Polyanion inducer concentrations were calculated from the midpoint concentration for successful amyloid induction in past studies^17^ and are presented in Supplementary Table 1. All inducer stocks and buffer were freshly prepared each day. After 48 h, reactions were ultracentrifuged at 103,000 × *g* using a tabletop Beckman Optima Max-XP Ultracentrifuge with TLA-55 rotor to separate fibrils from excess reaction components (for example, unreacted monomer, soluble oligomers). Pellets containing tau fibrils were resuspended in tau buffer, tested for positive ThT fluorescence to validate the presence of amyloid, and quantified using A_205_ absorbance on a Nanodrop Microvolume Spectrophotometer (Thermo Fisher Scientific).

To validate whether some of these fibrils might have distinct structures, we performed partial proteolysis experiments. Specifically, the fragments remaining after trypsin treatment were separated by SDS-PAGE and blotted with antibodies (named tau 13, tau 1, tau 5 and 4R) that recognize four different antigenic sites (Supplementary Fig. 1 and ref. ^37^). This proteolysis-enabled profiling approach is commonly used to reveal which epitopes in tau are relatively included/excluded from the fibril core^69^ and WT 0N4R tau monomer and fibrils have already been extensively characterized by this method^29^. As a control, we confirmed that tau P301S monomer appeared at the expected molecular weight of ~55 kDa in the absence of trypsin and that all four epitopes were completely hydrolysed by enzyme addition (Supplementary Fig. 1a). As expected, fibrils formed from WT and P301S tau, both incubated with the same inducer (that is, heparin), yielded distinct patterns^27,29^, consistent with them being different polymorphs. Similarly, varying the identity of the polyanion in the presence of P301S tau also tended to produce a variety of patterns, which we grouped into approximate categories based on epitope availability (Supplementary Fig. 1b).

### Purification of α synuclein protein

*Escherichia coli* BL21 Rosetta 2 (DE3) cells were transformed with a PET28a (+) vector containing full-length WT α synuclein and plated on a kanamycin resistant agar plate. An overnight culture was prepared from a single colony (20 ml per 1 l culture). Cells were grown to 0.8 optical density (OD_600_) in Luria broth (LB) + 50 µg ml^−1^ kanamycin, and then expression was induced with 0.4 mM IPTG for 4 h at 37 °C. Bacteria were pelleted by centrifugation (4,000 × g, 20 min, 4 °C) and then resuspended in 25 mM Tris-HCl, 0.1 mM PMSF pH 7.0 and EDTA free Complete protease inhibitor (15 ml per 1 l expression). Resuspended cells were lysed by sonication and the lysate was clarified by centrifugation (25,000 × g, 30 min, 4 °C). The mixture was boiled (15 min, 80 °C), and the precipitated impurities were removed by centrifugation (25,000 × g, 30 min, 4 °C). The lysate was treated with streptomycin (10% w/v, 1 ml per 1 l culture), acidified to pH 2–3 with 2 M HCl. The precipitated nucleic acids and proteins were removed by centrifugation (25,000 × g, 30 min, 4 °C). The buffer was exchanged with 25 mM Tris-HCl pH 8.0 with 3500 MWCO snakeskin dialysis tubing overnight. The material was purified over DEAE Sepharose ff (0–500 mM NaCl, 50 mM increments of 30 ml buffer, 30 ml resin bed). The fractions with α synuclein were dialysed into 10 mM NH_4_HCO_3_ with 3500 MWCO snakeskin dialysis tubing overnight and lyophilized. The material was dissolved in 3 ml of 6 M GndHCl, and purified over a C18 column (10 × 250 mm, 5 µM, 100 Å, 5–65% MeCN/H_2_O/0.1% TFA over 60 min). The pure α synuclein was lyophilized to afford ~10 mg per liter expression.

### Creation and purification of α synuclein fibrils

Aggregation reactions were performed in 1.5 ml Eppendorf tubes at 37 °C with constant agitation at 600 r.p.m. All reactions contained 300 µl of 500 µM α synuclein in 50 mM Tris-HCl, 150 mM KCl, 0.05% NaN_3_ pH 7.5. After 96 h the fibrils were centrifuged (20 × g, 30 min, 4 °C). Pellets containing α synuclein fibrils were resuspended in 300 µl PBS, aliquoted, snap-frozen and thawed before use.

### Synthesis of IAPP and preparation of IAPP fibrils

Wild-type and S20G IAPP were chemically synthesized complete with C-terminal amidation and the Cys2-Cys7 disulfide, as previously described^40^ IAPP was purified by reverse-phase high-performance liquid chromatography (Kinetex EVO C18 Column, Phenomenex) using a gradient of acetonitrile with 0.1% (v/v) formic acid. The identity and presence of the correct chemical modifications were confirmed by high-resolution mass spectrometry (masses 3903.3 Da for WT IAPP and 3873.3 Da for S20G), and the purity was assessed to be >95% by analytical high-performance liquid chromatography. After purification, peptide was lyophilized and stored at –20 °C until use.

IAPP fibrils were prepared in the same manner as described^41,42^. In summary, lyophilized WT or S20G IAPP was resolubilized by incubation in hexafluoroisopropanol (Sigma) at 1 mg ml^−1^ peptide for 30 min at room temperature, then aliquoted into 1.5 ml glass vials. The solvent was completely evaporated by swirling under a gentle stream of nitrogen gas to leave a peptide film. Peptide films were frozen for storage and thawed at room temperature (5 min) at the point of use. Fibrils were prepared by dissolving hexafluoroisopropanol-treated IAPP at 30 µM peptide in 20 mM ammonium acetate (pH 6.8), filtering the solution using a SpinX nitrocellulose-membrane filter (Corning) with centrifugation at 10,000 *g* for 5 min, and transferring to a clean glass vial. Vials were incubated for 2 yr (WT) or 1 yr (S20G), during which time IAPP monomers completely convert into amyloid fibrils^41,42^. The formation of amyloid fibrils was confirmed by negative stain TEM, and the morphology was consistent with our previous observations under those conditions^41^. Fibril samples were then snap-frozen until further use.

### paDSF screens

Tau fibrils (at least 100 µg ml^−1^) were screened for binding to 306 members of the Aurora dye library^20^. Briefly, screens were performed in 384-well plate format (Axygen) using a qTower real-time thermal cycler (Analytik Jena). Fibril stocks (2 µl) in tau buffer were added to individual wells, followed by the addition of dye (8 µl; final concentration between 0.5 to 50 µM, depending on the optimal value determined previously^20^). These mixtures were incubated at room temperature for 10 min. Three control screens were also performed. The first samples contained dye, but no fibril (termed the “no protein control”). The second contained dye and polyanion (termed polyanion-only control). The third samples contained monomeric tau (1 µM) without the polyanion inducer (termed the tau monomer control). Fluorescence was monitored for six excitation/emission wavelength pairs: 470 nm/520 nm (FAM), 515 nm/545 nm (JOE), 535 nm/580 nm (TAMRA), 565 nm/605 nm (ROX), 630 nm/670 nm (Cy5), and 660 nm/705 nm (Cy 5.5). Fluorescence was measured as a function of temperature, with a temperature ramp of 1 °C increase per cycle from 25 to 95 °C over the course of 1 h.

### paDSF data analysis

Potential candidates for dye-fibril interaction were identified based on fluorescence profiles. Specifically, we sought to identify samples with high initial fluorescence, followed by diminished signal intensity as temperature increased (presumably due to thermal melting or re-arrangement of the binding site(s)). To identify the top performing dyes, a Python-based scoring function was used, and this scoring function is available in full in ref. ^37^. Briefly, the scoring function assigns a score from 0 to 10 and scores were assigned as follows.

First, a score of 10 was assigned when the maximum fluorescence was greater than 1,000 RFU, the minimum fluorescence was greater than –1,000 RFU, the ∆fluorescence after buffer subtraction was >2,500 RFU and the temperature at which the maximum fluorescence occurred was <55 °C. A score of eight was assigned when the maximum fluorescence was greater than 1,000 RFU, the minimum fluorescence was greater than –2,000 RFU, the ∆fluorescence after buffer subtraction was between 1,500 and 2,500 RFU, and the temperature at which the maximum fluorescence occurred was <55 °C. A score of five was assigned when the maximum fluorescence was greater than 1,000 RFU, the minimum fluorescence was greater than –2,000 RFU, the ∆fluorescence after buffer subtraction was between 1,000 and 1,500 RFU, and the temperature at which the maximum fluorescence occurred was <55 °C. A score of three was assigned when the maximum fluorescence was greater than 2,000 RFU, the minimum fluorescence was greater than –2,000 RFU, the ∆fluorescence after buffer subtraction was greater than 2,000 RFU, and the temperature at which the maximum fluorescence occurred was between 55 and 70 °C. Finally, a score of one was assigned when the maximum fluorescence was greater than 2,000 RFU, the minimum fluorescence was greater than –2000 RFU, the ∆fluorescence after buffer subtraction was greater than 2,000 RFU, and the temperature at which the maximum fluorescence occurred was > 70 °C. All other fluorescence profiles received a score of zero.

All dye-fibril combinations that scored a ten were manually inspected to remove false positives. The most common artefact resulted from bright dyes, in which small pipetting/concentration differences seemed to result in large, but misleading, apparent RFU differences. Second-generation coumarin analogues (see Extended Data Fig. 1) were screened in the same manner with a concentration of 0.5 µM dye and scored manually as either hit or non-hit. For each of three replicates, hits were assigned a value of 1 (that is, a score of three indicates the second-generation dye was a hit in three independent screening experiments).

Overall, the assay performance was good, with a final, validated hit rate (<3%) that might be expected for screening campaign performed with a focused chemical library; however, we also noted that there was relatively poor reproducibility between biological replicates (<50%), with many dyes scoring well in one experiment and poorly in the other (see Fig. 1). Thus, one lesson learned from these experiments is that it might be important to leverage biological replicates early in the primary/secondary screening, perhaps using fibrils obtained from different patient samples or different brain regions from the same patient. Another important lesson was that multiple, independent secondary assays were needed to focus on the most robust fluoroprobes. Here, the EMBER assay seemed particularly good at identifying dyes that discriminate between polymorphs, while the ThT-like assays provided initial estimates of whether the fibril structure forms early or late in the aggregation process.

### Screen of α-synuclein fibrils

Pre-formed fibrils (2 µl) were mixed with Aurora dyes (10 µM; 8 µl) in 384-well microtiter plates, similar to the method used for tau and IAPP. As above, control wells included monomeric α-synuclein protein alone (100 µg ml^−1^) or dye alone (10 µM); however, for these experiments, fluorescence was measured at the individual dye-emission maxima (*E*_m_) in a SpectraMax M5 plate reader and the fibril concentration was varied (xxx µM). An increase in dye fluorescence in the presence of increasing fibril, but not monomer, by at least 3 s.d. was interpreted as active. Experiments were repeated three times in biological replicates (for example, fresh α-synuclein fibrils) and compounds that were active in all three experiments were considered putative hits for subsequent validation. These results were manually inspected to remove potential false positives (for example, linear fluorescence, unusual dose dependence). This procedure yielded a similar hit rate to the paDSF screens (Fig. 4a), suggesting that either approach might be used depending on equipment availability.

### EMBER confocal microscopy

The 27 dyes hits that passed initial screening steps described above were validated for direct binding to fibrils via fluorescence confocal microscopy imaging. For initial imaging, WT and P301S fibrils induced with heparin, sodium alginate and sodium hexametaphosphate (six fibril types) were deposited in individual wells of a Corning BioCoat 384-well, collagen type I-treated, flat-bottom microplate. The plate was centrifuged at 50 × g to deposit fibrils on the bottom of the plate. Dyes were diluted to screening concentrations (0.5 to 50 µM) in tau buffer (1× DPBS buffer (Corning, pH 7.4) with 2 mM MgCl_2_ and 1 mM DTT), centrifuged for 10 min at 17,000 × g to remove dye aggregates, and added to fibril-containing wells. If dye binding to fibril occurred, fibrils were imaged using our spectral confocal microscopy method, called EMBER imaging^22^. Fibrils stained with each active dye were imaged using a Leica Microsystems SP8 confocal microscope equipped with a 40× water-immersion lens (1.1 NA) and utilized a white light and 405 nm lasers along with a HyD detector to capture images at a resolution of 512 × 512 pixels at 1× zoom. The optical plane was autofocused with the highest sensitivity setting for each field of view. To minimize background noise, the LightGate was set between 0.5–18 ns. Initially, 110 images were captured using the Λ/λ-scan mode with wavelengths ranging from 470 nm to 670 nm at 20 nm intervals. The emission detection started at 10 nm above the given excitation wavelength and concluded at 780 nm within a 20 nm window. For instance, for the 470-nm excitation, images were collected from 480 nm to 780 nm at 20 nm intervals. Subsequently, in the *λ*-scan mode, 18 more images were captured at 405 nm excitation with emission detection intervals of 20 nm, ranging from 420 nm to 780 nm.

### EMBER conformational analysis of inducer-generated fibrils

The EMBER analytical pipeline to discriminate conformational strains of fibrils is described previously^22^. In brief, we developed a set of MATLAB scripts to process raw fluorescence images and to segment fibril particles from the background. The scripts yielded a particle-resolution EMBER profile for all of the recognized particles in each confocal experiment. We then normalized each identified particle-resolution EMBER spectra and concatenated EMBER profiles from both WT and P301S 0N4R tau fibrils corresponding to tested polyanions and specific dye. The data were then subjected to dimension reduction using principal component analysis. We implemented a quadratic fit discriminant analysis classifier (fitcdiscr) to quantify the discrimination power of a specific dye against WT and P301S 0N4R tau fibrils. The accuracy scores from this analysis served as the discrimination score, which were averaged across polyanions per specific dye to calculate an overall discrimination score (Fig. 3).

### Calculation of molecular similarities

Tanimoto similarities of all pairwise dye hit combinations were calculated using the RDkit Python package^70^.

### Real-time kinetic fibril detection assays (ThT-like assay)

Aggregation assays were performed in 384-well microplates (Corning 4511) coated with 0.01% Triton-X detergent. Wild type or P301S tau (10 µM) was combined with 50 µM dye (L016, L017, L033, L063, MWC034, MWC061, L079, L089 and L095), 5 µM dye (L031) or 10 µM ThT and freshly prepared polyanion inducer (refer to Supplementary Table 1 for the concentrations) in individual wells to a final volume of 20 µl in tau buffer (1× DPBS buffer (Corning, pH 7.4) with 2 mM MgCl_2_,1 mM DTT). Aggregation reactions were performed in a SpectraMax M5 microplate reader (Molecular Devices) at 37 °C with continuous shaking for 48 h. Fluorescence was monitored at four excitation/emission wavelength pairs: 444 nm/482 nm (cutoff 475 nm); 470 nm/520 nm (cutoff 515 nm); 515 nm/545 nm (cutoff 530 nm); and 565 nm/605 nm (cutoff 590 nm). Each reaction was performed in triplicate. To facilitate comparison of dyes between different reactions, RFU values were normalized between 0 and 1. We did not test all of the tau fibril samples in these experiments and, rather, focused only on the samples that had shown the most promising results in the EMBER studies.

Although the ThT-like assays increased our confidence that our paDSF platform yields useful chemical tools, it is possible that the presence of the fluoroprobes in the reaction could act as aggregation inhibitors/inducers or could influence the final fibril structure. Moreover, potential photophysical artefacts can make it challenging to decouple true kinetics from probe-induced effects. Indeed, dye hit L095, which robustly bound to mature fibrils in vitro and in histopathology samples, seemed to inhibit tau fibrillization across all inducers tested, perhaps because L095 binds to the site of monomer addition on a growing fibril. Thus, the performance of paDSF hits in downstream applications needs to be carefully considered.

### Ex vivo imaging of tau pathology in mouse brain

All animals were housed in a facility accredited by the Association for Assessment and Accreditation of Laboratory Animal Care International, in accordance with the Guide for the Care and Use of Laboratory Animals. All of the procedures for animal use were approved by the University of California, San Francisco’s Institutional Animal Care and Use Committee. Tg4510 mice express human MAPT containing the P301L mutation under the control of the forebrain-specific Ca^2+^/calmodulin kinase II promoter^71^. Brains from 18-month-old mice were harvested, immersion-fixed in 10% buffered formalin and then embedded in paraffin following standard procedures. The formalin-fixed and paraffin-embedded mouse brains were sectioned (8 μm thickness) and mounted on glass. To reduce the autofluorescence in the brain tissue, we photobleached the sections for up to 48 h in a cold room using a multispectral light-emitting diode array^72^. The sections were then deparaffinized and subjected to hydrolytic autoclaving at 105 °C for 20 min in citrate buffer (Sigma, C9999). Following blocking with 10% normal goat serum (Vector laboratories, S-1000), sections were incubated with primary antibodies, either with AT8 antibody (1:250 dilution; Thermo MN1020) followed by secondary Alexa Fluor goat anti-mouse 488 antibody (1:500 dilution; Thermo Fisher A11029) or with anti-tau phospho S396 antibody [EPR2731] (Abcam ab109390) 1:500 and Purified anti-β-Amyloid, 17–24 antibody clone 4G8 (Biolegend 800702) 1:1000, overnight at room temperature. After washing, sections were incubated in secondary antibodies Alexa Fluor goat anti rabbit 488 (Thermo Fisher A11008) and Alexa Fluor goat anti-mouse 405 (Thermo Fisher A48255) both 1:500 for 120 min at room temperature. Sections were then washed in PBS and incubated in L095 (50 µM) for 20 min and then rinsed with deionized water and coverslipped using Permafluor aqueous mounting medium (Thermo Fisher Scientific, TA030FM). Brain sections were imaged using a Leica Microsystems SP8 confocal microscope equipped with a 40× water-immersion lens (1.1 NA) and used a white light and 405 nm lasers along with a HyD detector to capture images at a resolution of 512 × 512 pixels at 2× zoom. To minimize background noise, the LightGate was set between 0.5–18 ns. Human brain tissue samples (Supplementary Table 3) were labelled similarly.

### Reporting summary

Further information on research design is available in the Nature Portfolio Reporting Summary linked to this article.

## Online content

Any methods, additional references, Nature Portfolio reporting summaries, source data, extended data, supplementary information, acknowledgements, peer review information; details of author contributions and competing interests; and statements of data and code availability are available at 10.1038/s41557-025-01889-7.

## Supplementary information

Supplementary Information Supplementary Tables 1-3 and Figs. 1–10.

Reporting Summary

## Data Availability

The following information is available at Zenodo at 10.5281/zenodo.14994580 (ref. ^37^): a description of the Aurora chemical library, uncropped partial proteolysis blots, the full histopathology dataset, raw paDSF tau screening data, raw screening data from the α-synuclein and IAPP screens, raw ThT-like kinetic assay data, and raw data from the coumarin analogue screen. All other data is provided in the Supplementary Information.

## Code availability

The Python codes used for the paDSF tau screening and Tanimoto analysis are available in our repository at 10.5281/zenodo.14994580 (ref. ^37^).
