# Author Correction: High-throughput discovery of fluoroprobes that recognize amyloid fibril polymorphs

**DOI:** 10.1038/s41557-026-02190-x

**Published:** 2026-05-27

**Authors:** Emma C. Carroll, Hyunjun Yang, Wyatt C. Powell, Annemarie F. Charvat, Abby Oehler, Julia G. Jones, Kelly M. Montgomery, Anthony Yung, Zoe Millbern, Alexander I. P. Taylor, Martin Wilkinson, Neil A. Ranson, Sheena E. Radford, Nelson R. Vinueza, William F. DeGrado, Daniel A. Mordes, Carlo Condello, Jason E. Gestwicki

**Affiliations:** 1 Department of Chemistry, San José State University, San José, CA USA; 2 Institute for Neurodegenerative Diseases, University of California, San Francisco, San Francisco, CA USA; 3 Department of Pharmaceutical Chemistry, University of California San Francisco, San Francisco, CA USA; 4 Department of Biochemistry, Brandeis University, Waltham, MA USA; 5 Department of Textile Engineering, Chemistry and Science, North Carolina State University, Raleigh, NC USA; 6 Astbury Centre for Structural Molecular Biology, School of Molecular and Cellular Biology, Faculty of Biological Sciences, University of Leeds, Leeds, UK; 7 Department of Pathology, University of California San Francisco, San Francisco, CA USA; 8 Department of Neurology, University of California San Francisco, San Francisco, CA USA

**Keywords:** Screening, Sensors and probes

Correction to: *Nature Chemistry* 10.1038/s41557-025-01889-7, published online 14 August 2025.

In the version of this article initially published, in the Fig. 5a coumarins panel, there were errors where the labels now reading left-to-right as “L016 (Coumarin 481)” and “L017 (Coumarin 485)” appeared originally as “L017 (Coumarin 485)” and “L016,” while in the polymethines panel, there were graphical errors in the structures shown for L063 and L095. The labels and structures are now amended in the HTML and PDF versions of the article.

