# Supplementary material for: High-throughput discovery of fluoroprobes that recognize amyloid fibril polymorphs
Source: Nat Chem. 2025 Aug 14;17(10):1565–75. doi: 10.1038/s41557-025-01889-7 (PMC12491066; doi:10.1038/s41557-025-01889-7)
Supplement: Supplementary file 1 — Supplementary Tables 1-3 and Figs. 1–10. [file 41557_2025_1889_MOESM1_ESM.pdf]

nature chemistry

Supplementary information https://doi.org/10.1038/s41557-025-01889-7

# High-throughput discovery of fluoroprobes that recognize amyloid fibril polymorphs

In the format provided by the authors and unedited

Supplementary Materials for Carroll *et al.*

# Methods for high throughput discovery of fluoroprobes that Recognize amyloid fibril polymorphs

# Table of Contents

*Table S1 (Polyanions used)* .......... *3*
*Figure S1 (Limited proteolysis)* .......... *4*
*Figure S2 (Full screening data heat maps)* .......... *5*
*Table S2 (Dyes tested in confocal microscopy)* .......... *6*
*Figure S3 (α-synuclein confocal validation)* .......... *7*
*Figure S4 (IAPP confocal validation)* .......... *8*
*Figure S5 (Tanimoto coefficients of hits and tau probes)* .......... *9*
*Figure S6 (L031 dye kinetics with ThT)* .......... *10*
*Figure S7 (Low versus high background binding in tissues)* ..........*11*
*Figure S8 (Controls showing that L095 also binds to Abeta)*.......... *12*
*Figure S9 (A003 recognition of α-synuclein in tissues)*.......... *13*
*Figure S10 (Coumarin analog chemical structures)* .......... *14*
*Table S3 (Sources of postmortem human brain tissue samples)*.......... *15*

| Number | Polyanion name | CAS Number | Supplier | Effective Induction Concentration (μg/mL) |
|---|---|---|---|---|
| 1 | Heparin Sodium | 9041-08-1 | Santa Cruz Biotechnology | 100 |
| 2 | Sodium Polyphosphate | 10361-03-2 | | 1000 |
| 3 | Poly-L-Glutamate sodium salt | 26247-79-0 | Sigma | 500 |
| 4 | Fondaparinux sodium | 114870-03-0 | Sigma | 500 |
| 5 | Nadroparin calcium | 37270-89-6 | | 250 |
| 6 | Sodium alginate | 9005-38-3 | Sigma | 500 |
| 7 | Sodium hexametaphosphate | 68915-31-1 | | 500 |
| 8 | Polystyrene sulfonate | 25704-18-1 | | 62.5 |
| 9 | Poly(A) | 26763-19-9 | Sigma | 500 |
| 10 | Sodium Tripolyphosphate | 7758-29-4 | | 2000 |
| 11 | Chondroitin sulfate A sodium salt | 39455-18-0 | Sigma | 250 |
| 12 | Diadenosine pentaphosphate | 75522-97-3 | Sigma | 500 |
| 13 | Dermatan sulfate and oversulfated chondroitin sulfate | EPY0001321 (product code) | Sigma | 125 |

**Table S1. Summary of polyanion inducers used to generate WT and P301S tau fibril polymorphs used in this study.**

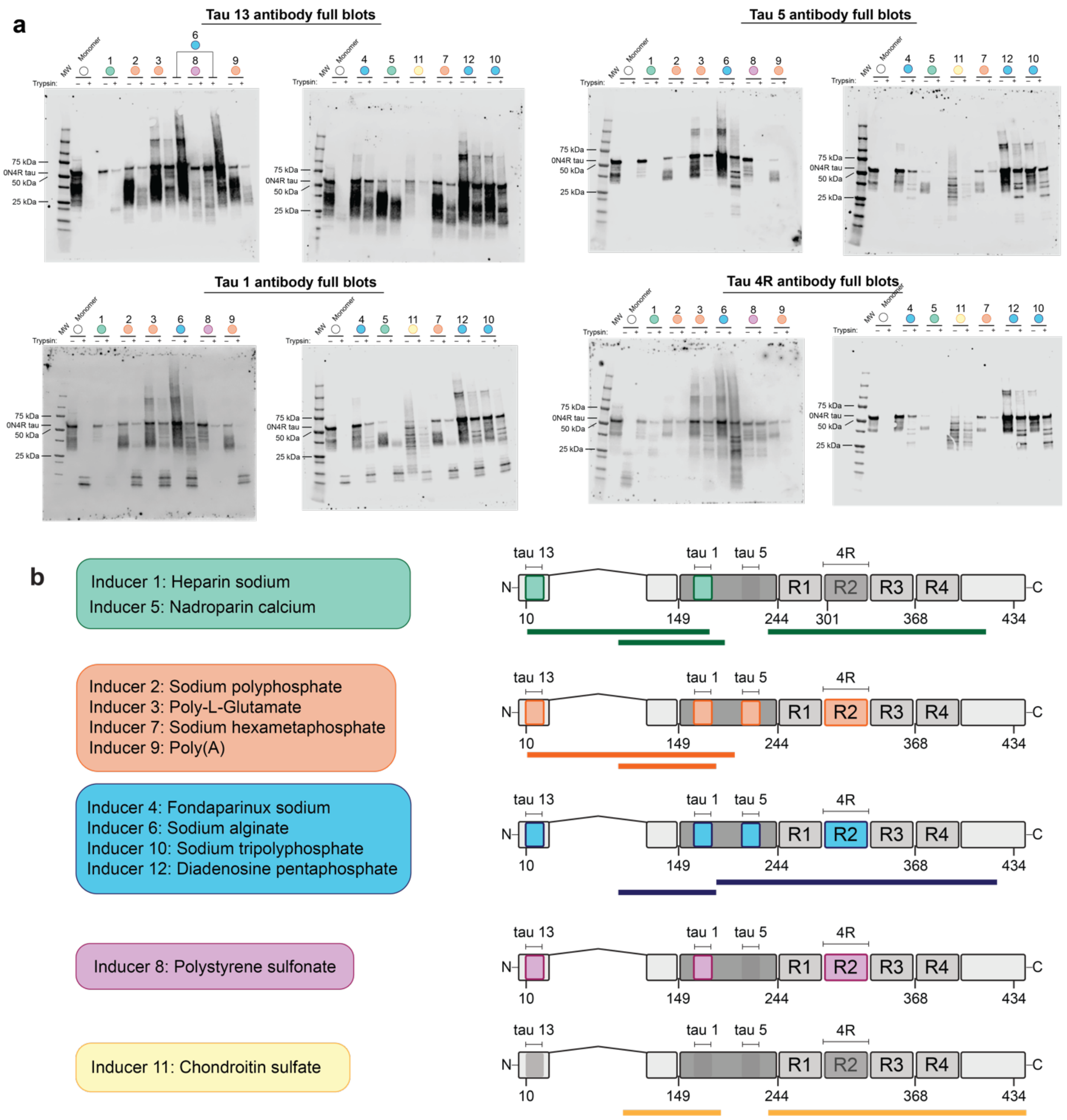


**Figure S1. Inducer-generated P301S tau fibrils also adopt diverse conformations that may be distinct from WT tau fibrils. (a)** Western blots with antibodies recognizing four different tau epitopes from limited proteolysis experiments performed with Promega sequencing grade trypsin at a fibril (or monomer):trypsin ratio 500:1 for 60 min. at 37 °C, as performed previously for WT tau in ref 29. The fibrils are generally resistant to trypsin cleavage compared to monomer, but yield distinct protease-resistant fragments that suggest considerable fibril conformational diversity among different inducers. **(b)** (Left) Categorization of polyanion-inducer generated fibrils that possess similar—but not necessarily identical—proteolysis patterns. (Right) Mapping of protease-resistant fragments to the 0N4R Tau P301S primary structure based on resistance of tau epitopes to cleavage for each category. Protease-resistant epitopes are shown in color with lines below the schematic representing general putative protease-resistant fragments observed within each category (not necessarily exact fragments for each inducer-generated fibril).

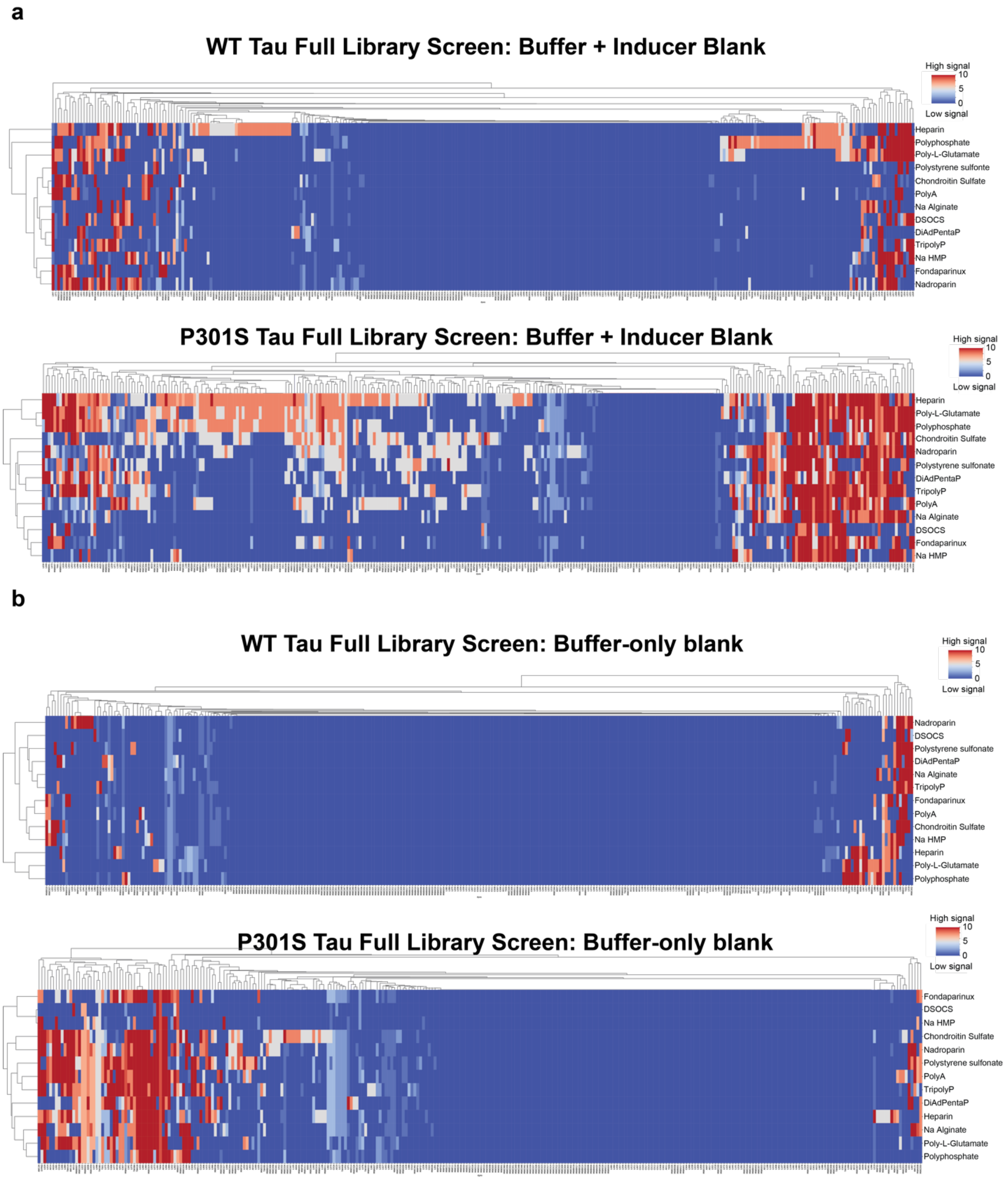


**Figure S2. Heat maps displaying full Aurora library screening data analyzed with different buffer blanks. (a)** Heat maps depicting scores generated from Python scoring function (see Extended data 2) of all Aurora library fluoroprobes incubated with 13 polyanion-induced WT tau fibrils (top) and P301S tau fibrils (bottom) using tau buffer (see methods) supplemented with the screening concentration (Table S1) of each polyanion **(b)** Heat maps depicting scores generated from Python scoring function (see Extended data 2) of all Aurora library fluoroprobes incubated with 13 polyanion-induced WT tau fibrils (top) and P301S tau fibrils (bottom) using tau buffer (see methods) alone.

| Dyes Tested for fibril binding using Confocal Microscopy | Binding Observed? |
|---|---|
| C018 | no |
| L009 | no* |
| L016 | yes |
| L017 | yes |
| L018 | no |
| L031 | yes |
| L033 | yes |
| L062 | no |
| L063 | yes |
| L073 | no |
| L077 | no |
| L079 | yes |
| L080 | no |
| L081 | no |
| L089 | yes |
| L095 | yes |
| L105 | no |
| MWA010 | no |
| MWA013 | no |
| MWC002 | no |
| MWC024 | no* |
| MWC027 | no |
| MWC034 | yes |
| MWC061 | yes |
| MWE07 | no |
| MWE08 | no |
| MWF03 | no |

*some potential binding observed but poor dye photophysics

**Table S2. Summary of 27 Aurora library fluoroprobes tested for direct binding to tau fibrils using confocal microscopy.** Yellow highlight indicates that a fluoroprobe is one of the ten final validated hits.

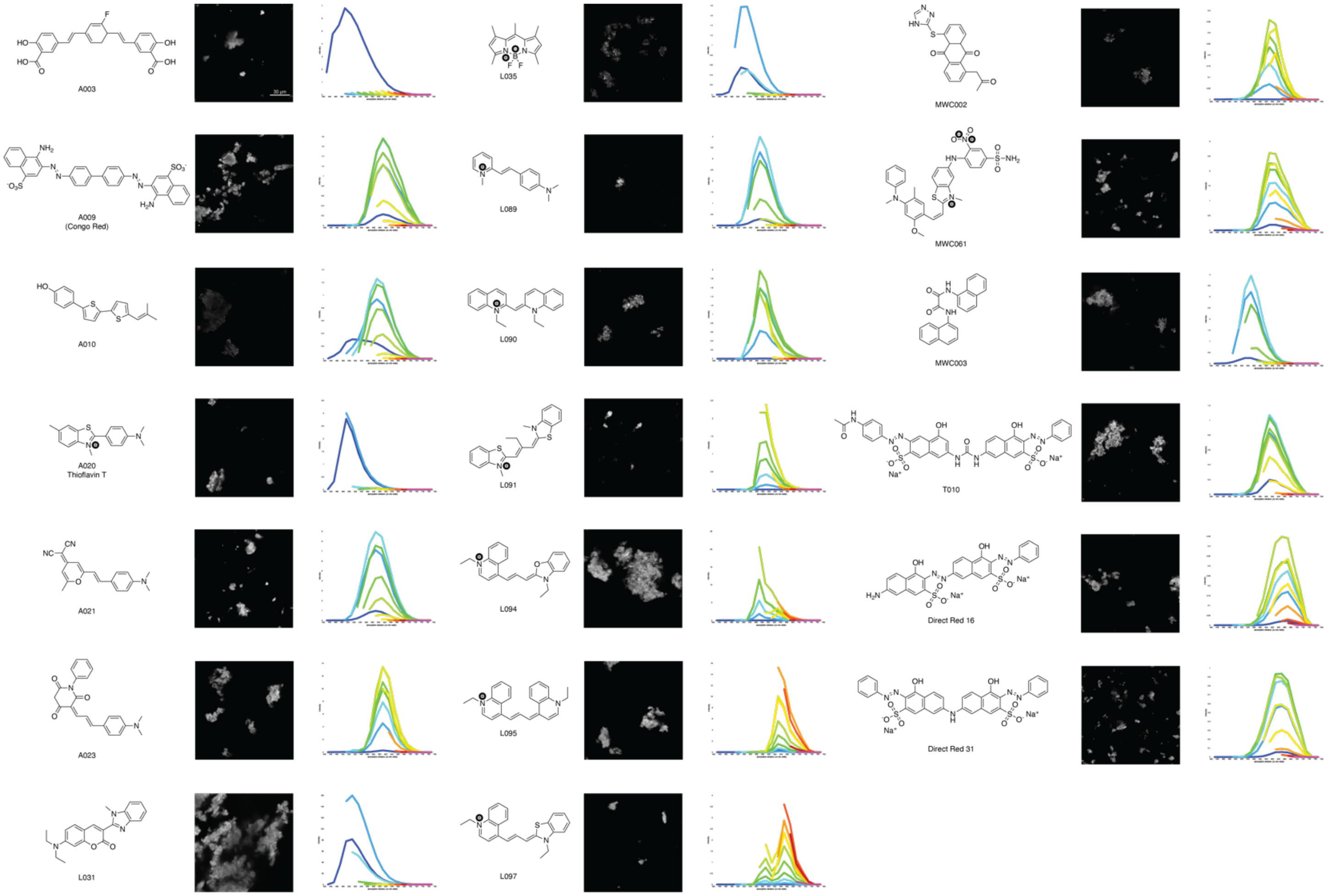


**Figure S3.** EMBER profiles and confocal micrographs of 20 hit dyes that each bound to synthetic α-Synuclein (α-Syn) fibrils. Initially, 33 hit dyes were identified from the primary screen (see Figure 4). 20 hit dyes demonstrated fluorogenic behavior upon binding to α-Syn fibrils in the EMBER assay. The plotted EMBER profiles represent the mean fluorescence intensity of segmented particles.

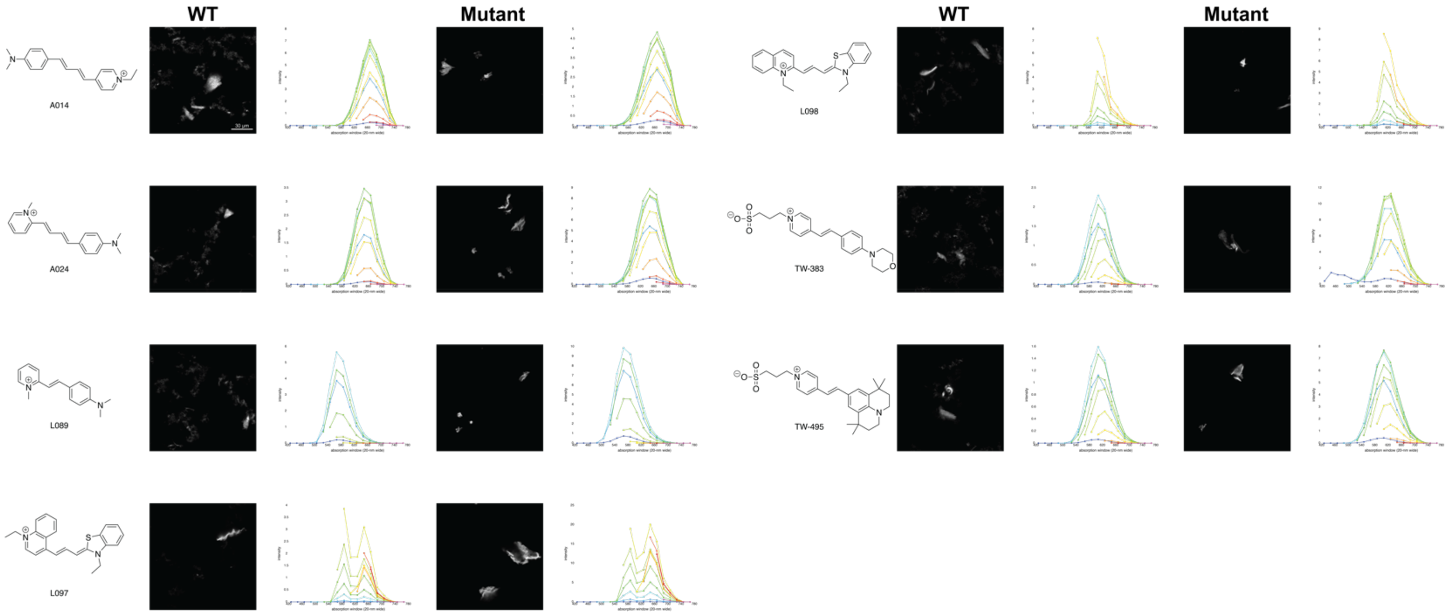


**Figure S4.** EMBER profiles and confocal micrographs of 7 hit dyes that each bound to wild-type (WT) and mutant islet amyloid polypeptide (IAPP) fibrils. Initially, 10 hit dyes were identified from the primary screen (see Figure 4). 7 hit dyes demonstrated fluorogenic behavior upon binding to WT and mutant IAPP fibrils in the EMBER assay. The plotted EMBER profiles represent the mean fluorescence intensity of segmented particles.

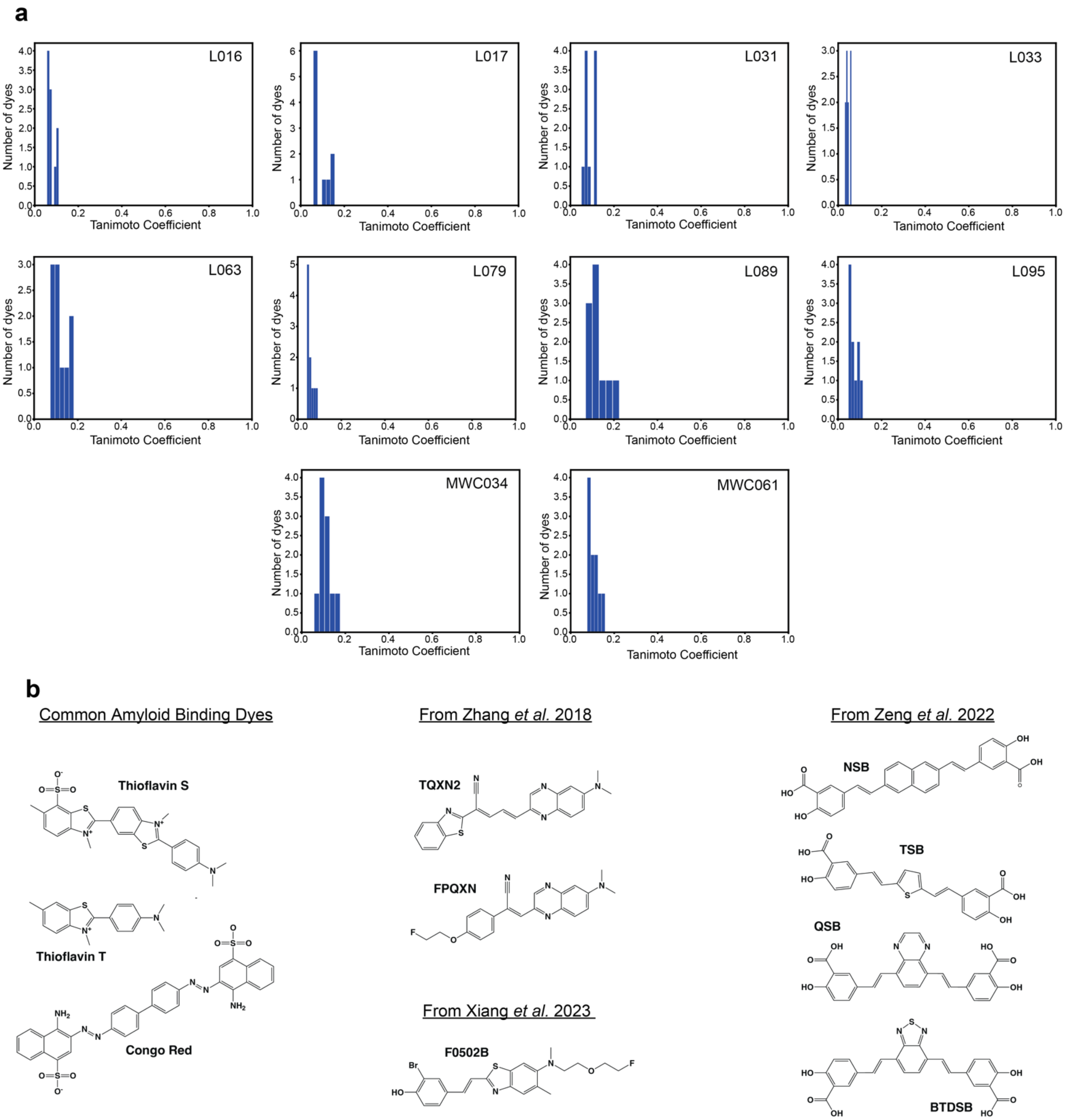


**Figure S5.** (a) Histograms reporting Tanimoto similarity coefficients (calculated with RDkit Python package) for all ten validated tau fibril-binding fluoroprobes and ten selected existing tau-binding probes from the literature. The small Tanimoto coefficients indicate that fluoroprobe hits from paDSF screens contain previously uncharacterized chemical scaffolds for tau fibril binding. (b) Full chemical structures and literature sources for all ten known tau-binding probes used to calculate Tanimoto coefficients with fluoroprobe hits.

Full references:

1. Xiang, J. et al. Development of an α-synuclein positron emission tomography tracer for imaging synucleinopathies. Cell 186, 3350-3367.e19 (2023).
2. Zeng, Q. et al. D-π-A-Based Trisubstituted Alkenes as Environmentally Sensitive Fluorescent Probes to Detect Lewy Pathologies. Anal. Chem. 94, 15261–15269 (2022).
3. Zhang, J. et al. Detection and Imaging of Aβ1-42 and Tau Fibrils by Redesigned Fluorescent X-34 Analogues. Chem. – Eur. J. 24, 7210–7216 (2018).

Dye hit L031 and Thioflavin T curves track closely across multiple inducers, perhaps due to a shared molecular rotor binding mode

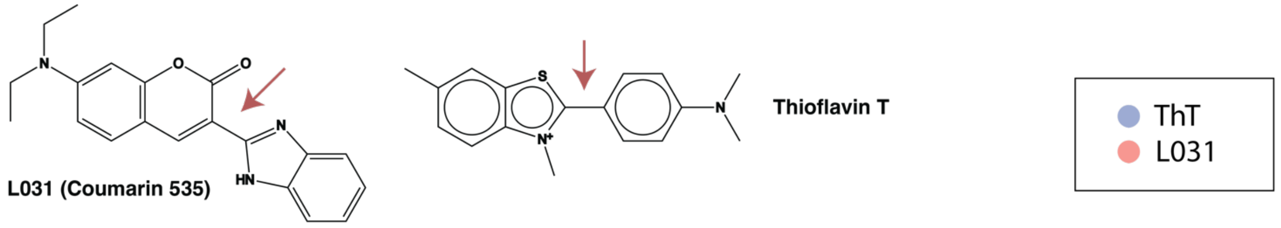


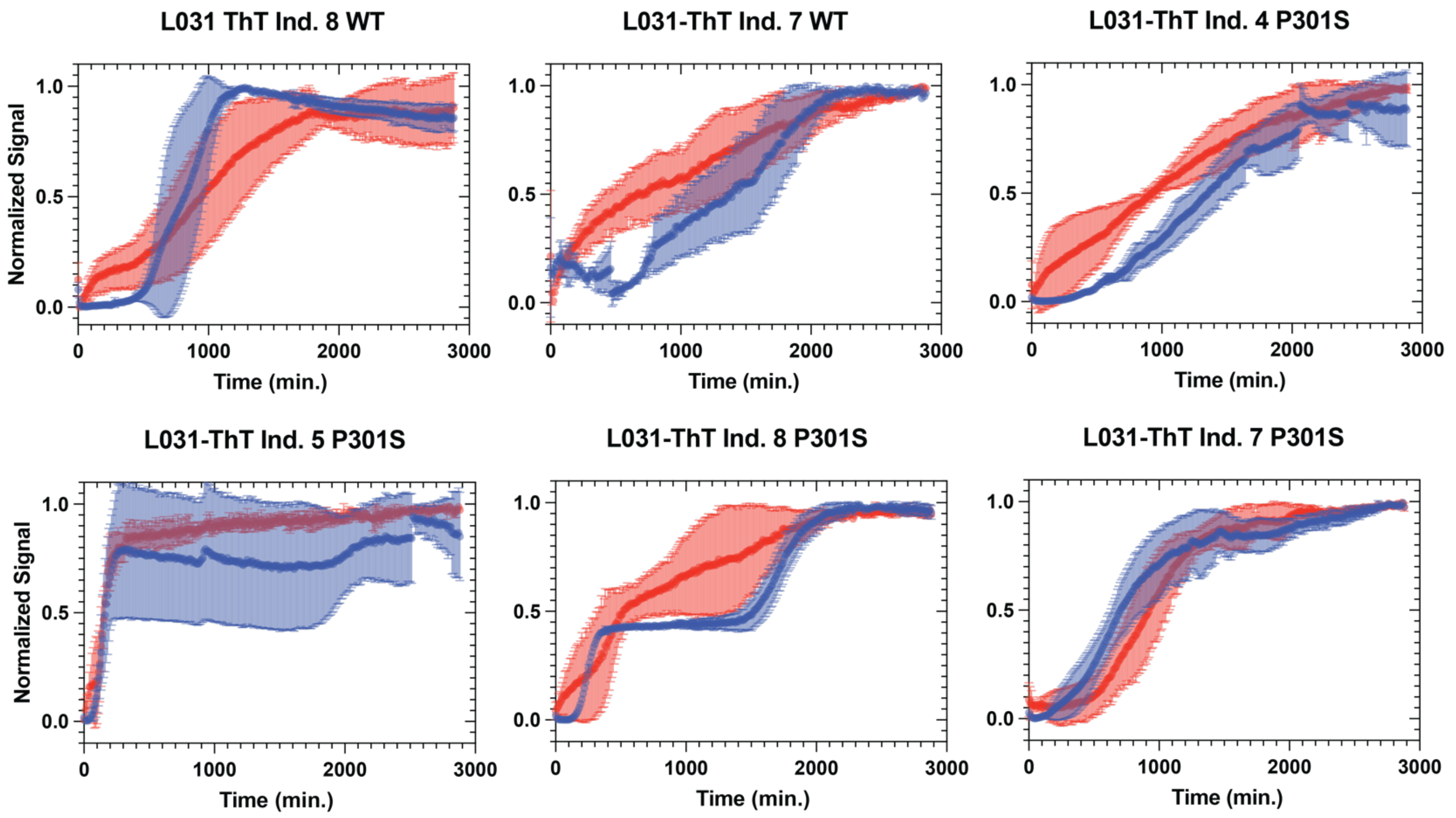


**Figure S6. Fluoroprobe L031 and Thioflavin T (ThT) curves track closely across multiple inducers, perhaps due to a shared molecular rotor binding mode.** Kinetic aggregation experiments performed with both WT and P301S tau and diverse polyanions reveal that the fluorescence profiles of dye L031 resemble those of ThT across multiple inducers. Thus, L031, which shares a molecular rotor architecture with ThT, may possess similar molecular recognition properties to (i.e., bind to similar binding sites during fibril formation) based on this close correspondence between their aggregation profiles. Both signals were monitored in the ThT channel (ex: 444, em: 482), n=3.

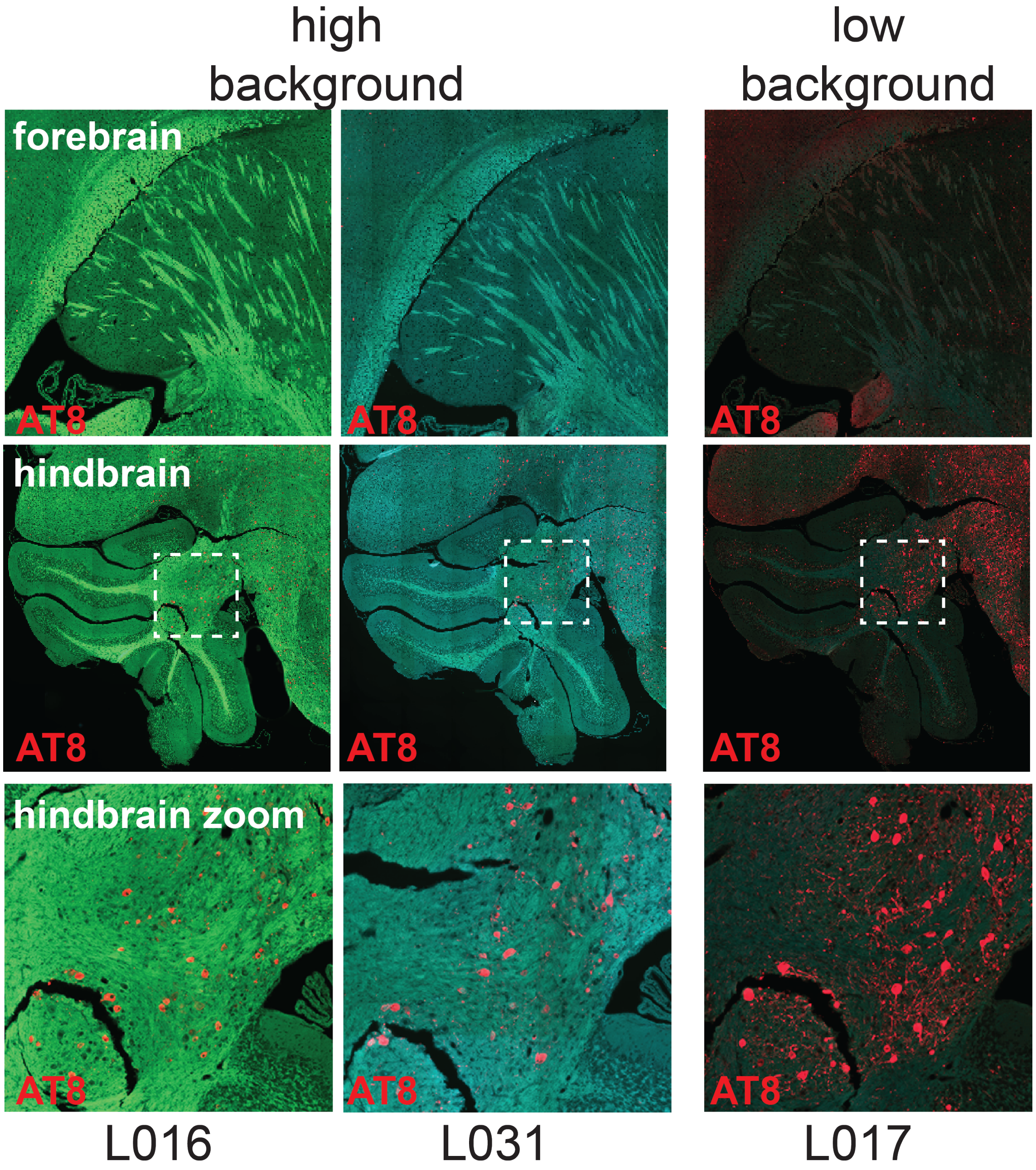


**Figure S7.** Scanned tg2541 brain slice images illustrating non-specific binding levels. L016 and L031 exhibit high background signals, whereas L017 shows low background. AT8 antibody staining is shown in red. Top: forebrain (e.g., cortex, corpus callosum, and striatum). Bottom: hindbrain (e.g., midbrain, cerebellum, and brainstem) with 4x zoom.

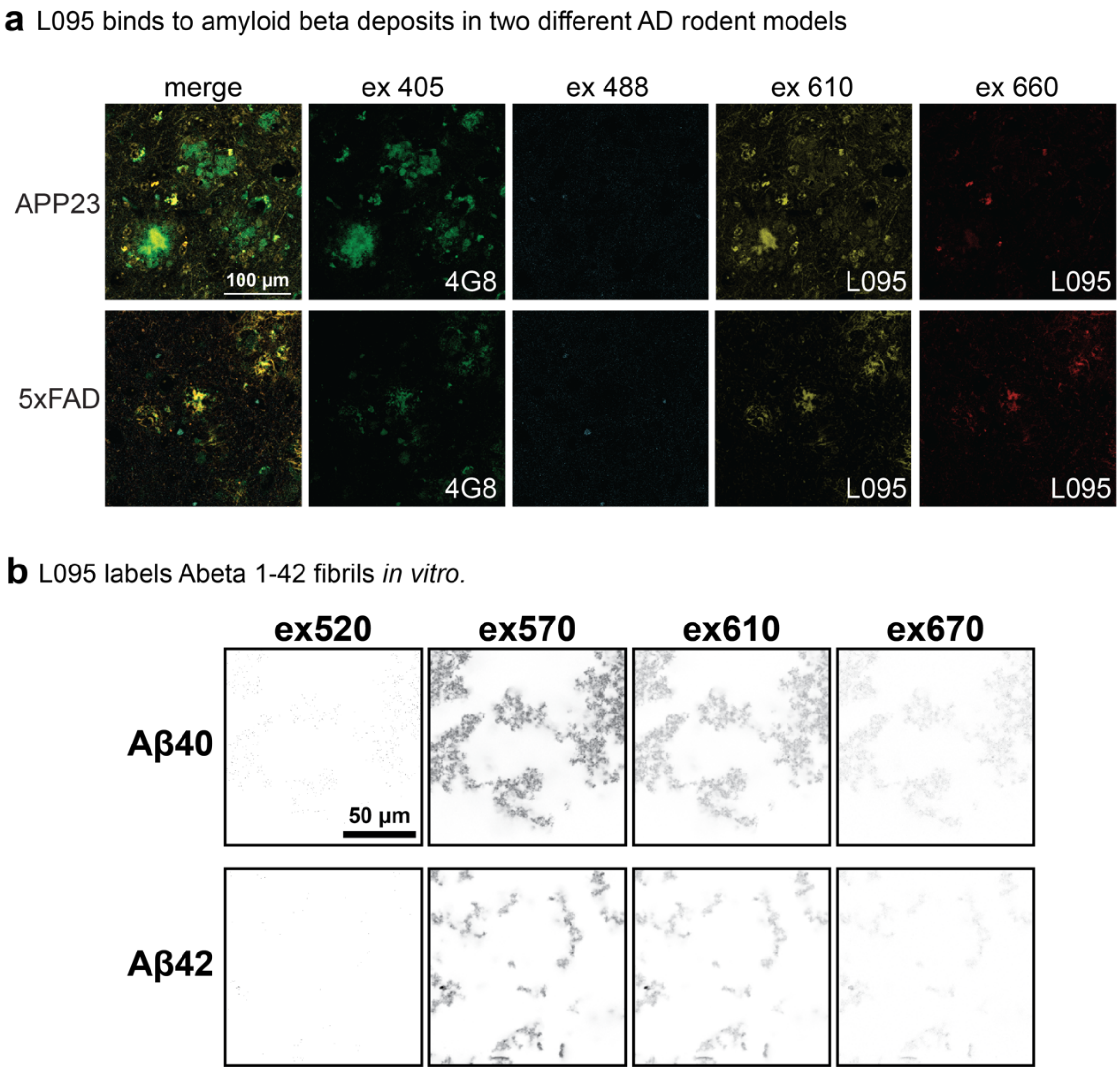


**Figure S8.** Control experiments show that dye L095 also binds to amyloid beta. (a) Staining of histology slides from two transgenic mouse models of Alzheimer's disease, showing overlap of dye L095 and an anti-amyloid beta antibody (4G8). Scale bar = 100 µm. (b) Binding of L095 to recombinant Abeta 1-42 fibrils, as measured by EMBER.

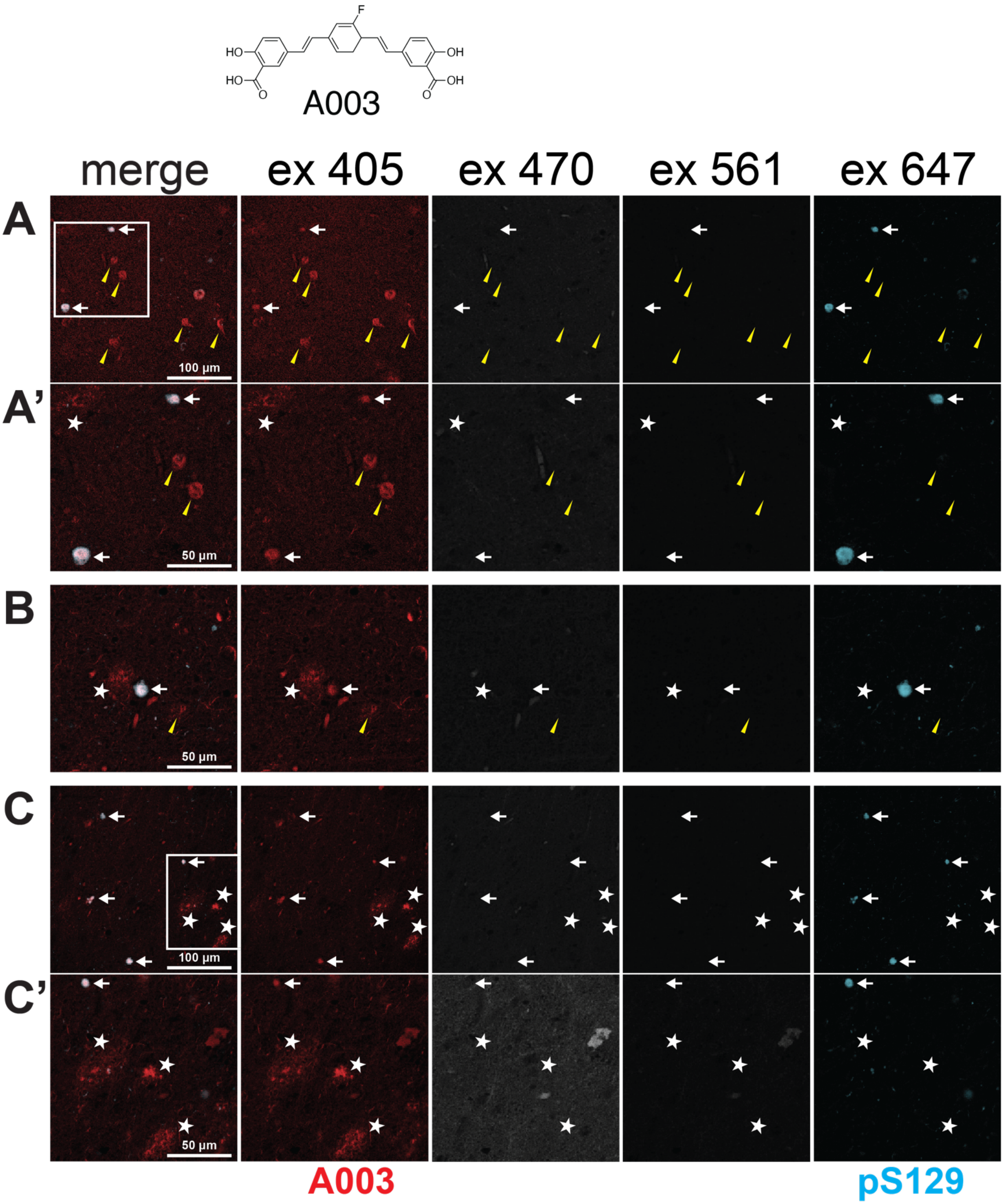


**Figure S9.** Fluoroprobe A003 (FSB) recognizes α-Synuclein (α-Syn) pathology in brain tissue from a patient with Dementia with Lewy Bodies (DLB). Representative micrographs are shown, with 2× zoom indicated by an apostrophe and a white rectangle box. The tissue was stained with a mouse monoclonal α-Syn phospho-S129 (pS129) antibody (blue; MABN826; Sigma-Aldrich) and A003 dye (red). Confocal images were acquired at four wavelengths (405 nm, 470 nm, 561 nm, and 647 nm). In the merged view, the signal from A003 at 405 nm co-localizes with the pS129 signal at 647 nm (white arrow). Note that A003 also detects Aβ plaques (white star) and tau tangles (yellow arrowhead).

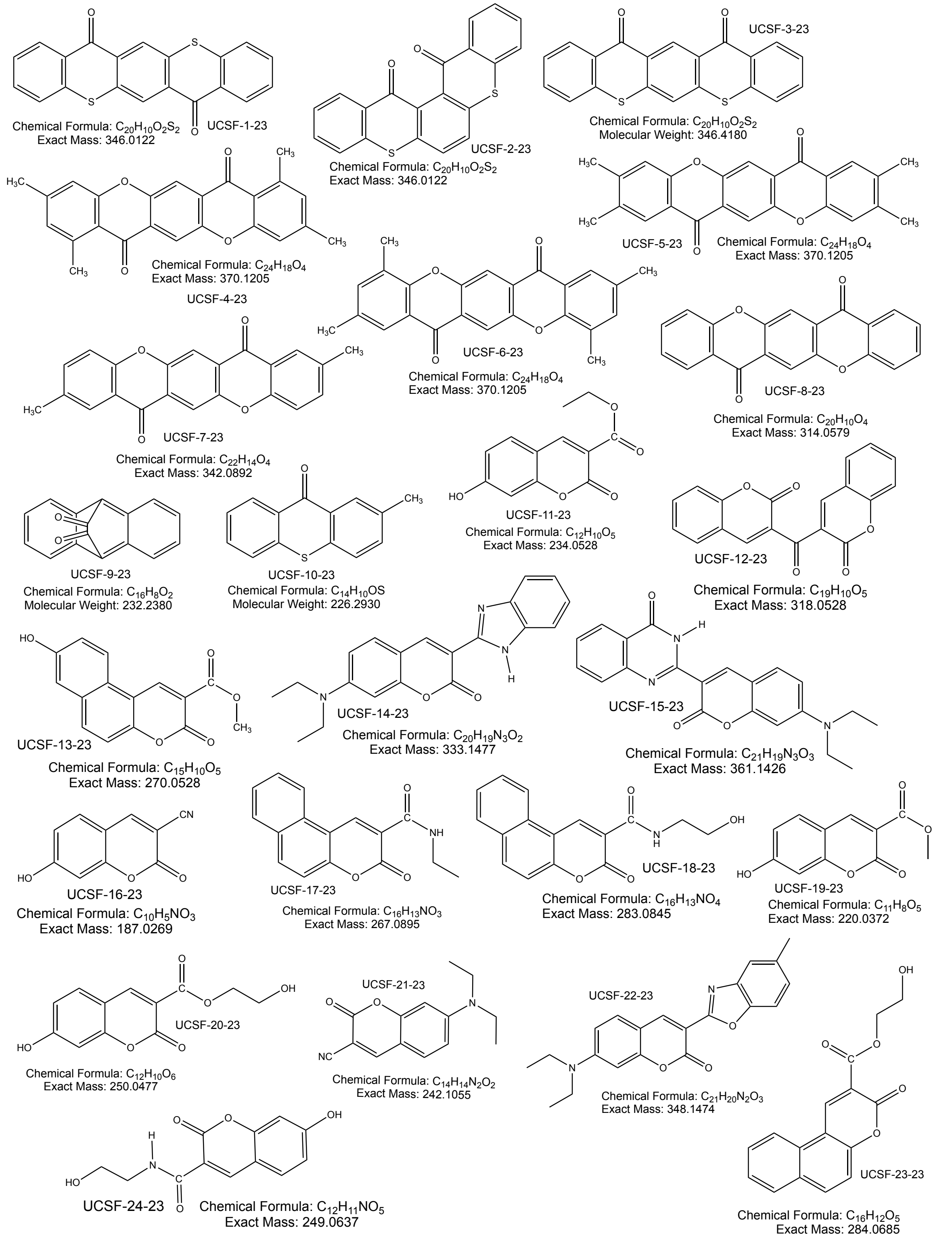


**Figure S10. Full chemical structures and formulas with molecular weights (in g/mol) for all coumarin-analog fluoroprobes discovered in a structural homology search of the Max Weaver Dye Library.** All compounds were screened with the 26 polyanion-induced fibrils in the second-generation paDSF screens.

| Cohort | Paitent ID # | Sex | Age | NPDx (Clinical Dx) | Mutation | APOE genotype | Cerad score | Braak stage | Brain region | Source |
|---|---|---|---|---|---|---|---|---|---|---|
| AD 1 | A2201 P2926B4 | M | 82 | AD | – | – | C3 | VI | Occipital | UCSF IND |
| AD 2 | BBN13829 A029/98x30 | M | 42 | AD | Δ4 PSEN1 | 3/3 | C3 | VI | Temporal cortex | King's College London (UK) |
| AD 3 | BBN13932 A0258/94 | F | 55 | AD | V717I APP (London) | 3/4 | C3 | VI | Temporal cortex | King's College London (UK) |
| DLB | 12-26 | M | 68 | DLB/AD | – | 3/4 | C3 | V | Medial Frontal Gyrus | Banner Sun Health |

**Table S3. Sources of postmortem human brain tissue samples.**
