# Supplementary material for: High-throughput discovery of fluoroprobes that recognize amyloid fibril polymorphs
Source: Nat Chem. 2025 Aug 14;17(10):1565–75. doi: 10.1038/s41557-025-01889-7 (PMC12491066; doi:10.1038/s41557-025-01889-7)
Supplement: Supplementary file 2 — Reporting Summary [file 41557_2025_1889_MOESM2_ESM.pdf]

nature portfolio

Corresponding author(s): Gestwicki, Jason E.

Last updated by author(s): Mar 10, 2025

# Reporting Summary

Nature Portfolio wishes to improve the reproducibility of the work that we publish. This form provides structure for consistency and transparency in reporting. For further information on Nature Portfolio policies, see our Editorial Policies and the Editorial Policy Checklist.

## Statistics

For all statistical analyses, confirm that the following items are present in the figure legend, table legend, main text, or Methods section.

| n/a | Confirmed | |
|---|---|---|
| [ ] | [x] | The exact sample size (*n*) for each experimental group/condition, given as a discrete number and unit of measurement |
| [ ] | [x] | A statement on whether measurements were taken from distinct samples or whether the same sample was measured repeatedly |
| [ ] | [x] | The statistical test(s) used AND whether they are one- or two-sided<br>*Only common tests should be described solely by name; describe more complex techniques in the Methods section.* |
| [x] | [ ] | A description of all covariates tested |
| [ ] | [x] | A description of any assumptions or corrections, such as tests of normality and adjustment for multiple comparisons |
| [ ] | [x] | A full description of the statistical parameters including central tendency (e.g. means) or other basic estimates (e.g. regression coefficient) AND variation (e.g. standard deviation) or associated estimates of uncertainty (e.g. confidence intervals) |
| [x] | [ ] | For null hypothesis testing, the test statistic (e.g. *F*, *t*, *r*) with confidence intervals, effect sizes, degrees of freedom and *P* value noted<br>*Give P values as exact values whenever suitable.* |
| [x] | [ ] | For Bayesian analysis, information on the choice of priors and Markov chain Monte Carlo settings |
| [x] | [ ] | For hierarchical and complex designs, identification of the appropriate level for tests and full reporting of outcomes |
| [x] | [ ] | Estimates of effect sizes (e.g. Cohen's *d*, Pearson's *r*), indicating how they were calculated |

*Our web collection on statistics for biologists contains articles on many of the points above.*

## Software and code

Policy information about availability of computer code

| | |
|---|---|
| Data collection | Molecular Devices software, Analytika Jena software |
| Data analysis | GraphPad PRISM, RDkit Python, MatLab, Google Colab |

For manuscripts utilizing custom algorithms or software that are central to the research but not yet described in published literature, software must be made available to editors and reviewers. We strongly encourage code deposition in a community repository (e.g. GitHub). See the Nature Portfolio guidelines for submitting code & software for further information.

## Data

Policy information about availability of data

All manuscripts must include a data availability statement. This statement should provide the following information, where applicable:

- Accession codes, unique identifiers, or web links for publicly available datasets
- A description of any restrictions on data availability
- For clinical datasets or third party data, please ensure that the statement adheres to our policy

The description of the Aurora library is found in Extended Data 1 (https://doi.org/10.5281/zenodo.13357986). The uncropped partial proteolysis blots are available in Extended Data 2 (https://doi.org/10.5281/zenodo.13357986). The raw paDSF tau screening data and associated Python code are available in Extended Data 3 (https://doi.org/10.5281/zenodo.14994580). The full histopathology dataset is available in Extended Data 4 (https://doi.org/10.5281/zenodo.13357986). The raw screening data from the [illegible]-synuclein and IAPP screens is in Extended Data 5 (https://doi.org/10.5281/zenodo.14994580). The raw ThT-like kinetic assay data and

Tanimoto analysis code are available in Extended Data 6 (https://doi.org/10.5281/zenodo.14994580). Raw data from the coumarin screen is in Extended Data 7 (https://doi.org/10.5281/zenodo.14994580). The raw EMBER data is available (https://datadryad.org/stash/share/YS5EBV9EomjU_vCXUm7-1Wzq23WTkVTyVUg3wuRgBA4 or doi:10.5061/dryad.s4mw6m9g4). All other data is provided in the Supplemental Information.

## Human research participants

Policy information about studies involving human research participants and Sex and Gender in Research.

| | |
|---|---|
| Reporting on sex and gender | *Use the terms sex (biological attribute) and gender (shaped by social and cultural circumstances) carefully in order to avoid confusing both terms. Indicate if findings apply to only one sex or gender; describe whether sex and gender were considered in study design whether sex and/or gender was determined based on self-reporting or assigned and methods used. Provide in the source data disaggregated sex and gender data where this information has been collected, and consent has been obtained for sharing of individual-level data; provide overall numbers in this Reporting Summary. Please state if this information has not been collected. Report sex- and gender-based analyses where performed, justify reasons for lack of sex- and gender-based analysis.* |
| Population characteristics | *Describe the covariate-relevant population characteristics of the human research participants (e.g. age, genotypic information, past and current diagnosis and treatment categories). If you filled out the behavioural & social sciences study design questions and have nothing to add here, write "See above."* |
| Recruitment | *Describe how participants were recruited. Outline any potential self-selection bias or other biases that may be present and how these are likely to impact results.* |
| Ethics oversight | *Identify the organization(s) that approved the study protocol.* |

Note that full information on the approval of the study protocol must also be provided in the manuscript.

# Field-specific reporting

Please select the one below that is the best fit for your research. If you are not sure, read the appropriate sections before making your selection.

☒ Life sciences ☐ Behavioural & social sciences ☐ Ecological, evolutionary & environmental sciences

For a reference copy of the document with all sections, see nature.com/documents/nr-reporting-summary-flat.pdf

# Life sciences study design

All studies must disclose on these points even when the disclosure is negative.

| | |
|---|---|
| Sample size | The sample sizes are described in each figure legend for each experiment. |
| Data exclusions | No data was excluded. |
| Replication | The number of replicates (both biological and technical) are included in each figure legend. In general, most experiments were performed in three independent biological replicates. |
| Randomization | All experiments use comparisons to specific controls, such as solvent controls or comparisons to known ligands. |
| Blinding | Blinding was not used in these studies, owing to the availability of positive and negative controls and the quantitative measurements used to make conclusions. |

# Reporting for specific materials, systems and methods

We require information from authors about some types of materials, experimental systems and methods used in many studies. Here, indicate whether each material, system or method listed is relevant to your study. If you are not sure if a list item applies to your research, read the appropriate section before selecting a response.

## Materials & experimental systems

| n/a | Involved in the study |
| --- | --- |
| ☐ | ☒ Antibodies |
| ☒ | ☐ Eukaryotic cell lines |
| ☒ | ☐ Palaeontology and archaeology |
| ☒ | ☐ Animals and other organisms |
| ☒ | ☐ Clinical data |
| ☒ | ☐ Dual use research of concern |

## Methods

| n/a | Involved in the study |
| --- | --- |
| ☒ | ☐ ChIP-seq |
| ☒ | ☐ Flow cytometry |
| ☒ | ☐ MRI-based neuroimaging |

# Antibodies

| | |
| --- | --- |
| Antibodies used | Antibodies are described in the Methods section. |
| Validation | No additional validation was performed. All antibodies are commercial and commonly used in the field. |
